# Acute *Toxoplasma* infection in pregnant women worldwide: A systematic review and meta-analysis

**DOI:** 10.1371/journal.pntd.0007807

**Published:** 2019-10-14

**Authors:** Ali Rostami, Seyed Mohammad Riahi, Despina G. Contopoulos-Ioannidis, H. Ray Gamble, Yadolah Fakhri, Malihe Nourollahpour Shiadeh, Masoud Foroutan, Hamed Behniafar, Ali Taghipour, Yvonne A. Maldonado, Ali H. Mokdad, Robin B. Gasser

**Affiliations:** 1 Infectious Diseases and Tropical Medicine Research Center, Health Research Institute, Babol University of Medical Sciences, Babol, Iran; 2 Immunoregulation Research Center, Health Research Institute, Babol University of Medical Sciences, Babol, Iran; 3 Cardiovascular Diseases Research Center, Department of Epidemiology and Biostatistics, Faculty of Health, Birjand University of Medical Sciences, Birjand, Iran; 4 Department of Pediatrics, Division of Infectious Diseases, Stanford University School of Medicine, Stanford, California, United States of America; 5 National Academy of Sciences, Washington, DC, United States of America; 6 Department of Environmental Health Engineering, Student Research Committee, School of Public Health, Shahid Beheshti University of Medical Sciences, Tehran, Iran; 7 Sexual and Reproductive Health Research Center, Mazandaran University of Medical Sciences, Sari, Iran; 8 Department of Parasitology, Faculty of Medical Sciences, Tarbiat Modares University, Tehran, Iran; 9 Department of Parasitology and Mycology, School of Medicine, Shahid Beheshti University of Medical Sciences, Tehran, Iran; 10 Department of Health Research and Policy, Stanford University School of Medicine, Stanford, California, United States of America; 11 Department of Health Metrics Science and the Institute for Health Metrics and Evaluation, University of Washington, Seattle, Washington, United States of America; 12 Department of Veterinary Biosciences, Melbourne Veterinary School, The University of Melbourne, Parkville, VIC, Australia; Johns Hopkins Bloomberg School of Public Health, UNITED STATES

## Abstract

**Background:**

Acute *Toxoplasma* infection (ATI) during pregnancy, if left untreated, can cause severe adverse outcomes for the fetus and newborn. Here, we undertook a meta-analysis to estimate the worldwide prevalence of ATI in pregnant women.

**Methods:**

We searched international databases for studies published between January 1988 and November 2018. We included population-based cross-sectional and prospective cohort studies that reported the prevalence of ATI in pregnant women. Data were synthesized using a random effect model to calculate the overall prevalence of ATI (with a 95% CI) in six WHO regions and globally. We also performed linear meta-regression analyses to investigate associations of maternal, socio-demographic, geographical and climate parameters with the prevalence of ATI.

**Results:**

In total, 217 studies comprising 902,228 pregnant women across 74 countries were included in the meta-analysis. The overall prevalence of ATI in pregnant women globally was 1.1% (95% CI: 0.9–1.2%). In studies where more strict criteria for ATI were used, the overall prevalence was 0.6% (95% CI: 0.4–0.7%). The prevalence was highest in the Eastern Mediterranean region (2.5%; 95%CI: 1.7–3.4%) and lowest in the European region (0.5%; 95% CI: 0.4–0.7%). A significantly higher prevalence of ATI was found in countries with lower income levels (*P* = 0.027), lower human development indices (*P* = 0.04), higher temperatures (*P* = 0.02) and lower latitudes (*P* = 0.005) and longitudes (*P* = 0.02).

**Conclusions:**

The risk of acquiring ATI during gestation is clinically important and preventive measures to avoid exposure of pregnant women to *Toxoplasma* infection should be strictly applied.

## Author summary

Acute infection during pregnancy with the protozoan parasite *Toxoplasma gondii*, if left undiagnosed and untreated, is linked to a wide range of medical complications, and severe adverse outcomes for the infected fetus and newborn. This is the first comprehensive systematic review and meta-analysis of the global prevalence of ATI in pregnant women, and was based on 217 studies of a total of 902,228 pregnant women from 74 countries. The overall global prevalence of ATI was 1.1%. According to studies that used strict criteria (seroconversion and low IgG avidity) for the definition of ATI, 0.6% of pregnant women had ATI during gestation. Countries with high-income levels and higher human development indices had significantly lower prevalence of ATI. In addition, the prevalence of ATI varied among countries depending on geographical location and climatic features. The findings of this study should increase awareness among public health leaders and educators regarding the global burden of ATI in pregnant women—which translates to a significant number of children worldwide being at risk of acquiring congenital toxoplasmosis and developing long-term sequelae—if maternal infection is left undiagnosed and untreated. The findings also indicate a need for universal prenatal screening for early diagnosis and treatment.

## Introduction

Acute *Toxoplasma* infection (ATI) during pregnancy, if left undiagnosed and untreated, can result in congenital toxoplasmosis (CT), which can cause severe, and often life-threatening disease with significant morbidity and mortality of fetuses and newborns [1–3]. Globally, the annual incidence of CT is estimated to be 190,100 cases (179,300–206,300), accounting for 1.2 million disability-adjusted life years (DALYs) annually [3]. The spectrum of disease of CT is wide, and fetuses and infants with CT can be asymptomatic or can present with severe symptoms including cerebral calcification, hydrocephalus or microcephaly, seizures, developmental delays, chorioretinitis, strabismus, vision loss, hearing loss, hepatosplenomegaly, jaundice, petechiae, thrombocytopenia, anemia and/or transaminitis [4–6]. ATI during pregnancy can also be asymptomatic or can cause a mild, flu-like illness with low-grade fever, fatigue and lymphadenopathy. Without universal prenatal screening strategies, the majority of ATIs during pregnancy will remain undiagnosed and untreated [1, 7].

CT in the fetus occurs through transplacental transmission of *T*. *gondii* after a primary maternal ATI during gestation or close to conception. In some immunocompromised, pregnant women, who had been *T*. *gondii* seropositive before pregnancy and not on anti-*Toxoplasma* prophylaxis, mother-to-child transmission (MTCT) can also occur from reactivation of a latent infection. Several factors affect the risk of MTCT, including the gestational age (GA) at the time of ATI [8], the virulence of the parasite strain or genotype, the parasite load during ATI and the delay in initiation of treatment following acute maternal infection. Early detection of ATI and prompt initiation of appropriate treatment reduces MTCT and ameliorates the severity of the disease in the fetus and the newborn [8–14]. Routine serological prenatal screening throughout gestation is important for early diagnosis and treatment of ATI during pregnancy [15, 16]. The diagnosis of ATI during pregnancy can be made based on (a) no detectable serum IgG anti-*Toxoplasma* antibodies, but detection of specific IgM, and/or IgA, and/or IgE antibodies [1, 17–19]; (b) detection of low avidity serum anti-*Toxoplasma* IgG antibodies; or (c) seroconversion from IgG negative to IgG positive status (usually combined with detection of anti-*Toxoplasma* IgM antibodies) in cases of sequential testing during gestation. The IgG avidity test, which measures the affinity of IgG antibody binding to *T*. *gondii* antigens, is low during the acute stages of infection and becomes high as the infection progresses to the chronic stage [20, 21]. Low IgG avidity can distinguish between *T*. *gondii* infection acquired < 12–16 weeks vs. > 12–16 weeks from the time of testing, and is particularly useful for the differentiation of acute from chronic maternal infections early in gestation. Seroconversion during gestation is considered the best indicator of ATI, and it can be used also to estimate the most likely time of ATI [1, 19, 22].

Despite a large number of published epidemiological studies in different countries, estimates of the regional and global prevalence of ATI in pregnant women are lacking. Except for some Western European countries, where mandatory monthly or bimonthly prenatal serological screening programs for *Toxoplasma* infection in pregnant women are implemented, screening for *Toxoplasma* infection during pregnancy is either not done, or is done only once at the beginning of pregnancy or once during each trimester. Awareness about the potential devastating sequelae of ATI during pregnancy remains low, even in countries with a high burden of toxoplasmosis. Empirical evaluation of the regional and global prevalence of ATI in pregnant women can help increase awareness among health-care policymakers and assist in developing guidelines to address this serious public health issue, including implementation of prenatal screening and treatment programs for *Toxoplasma* infection. To that end, we performed a systematic review and meta-analysis to estimate the worldwide prevalence of ATI in pregnant women. Furthermore, we performed ecological meta-regression analyses to evaluate the impact of maternal, geographical, climate and sociodemographic factors on the prevalence of ATI in different regions and countries.

## Methods

This meta-analysis was performed and reported in accordance with the Preferred Reporting Items for Systematic Reviews and Meta-analyses (PRISMA) guidelines [23]. The protocol for this meta-analysis was registered in PROSPERO (CRD42018108025).

### Search strategy and selection criteria

Two independent investigators (M.N.S. and M.F.) systematically searched PubMed/MEDLINE, Web of Science, EMBASE, Scopus and SciELO from 1 January 1988 to 30 November 2018. Employing the Boolean operators “OR” and/or “AND”, the following search terms were used in our literature searches: “*Toxoplasma gondii*”, “*Toxoplasma* infection”, “toxoplasmosis”, “acute toxoplasmosis”, “seroconversion”, “primary infection”, “seroepidemiology”, “seroprevalence”, “incidence”, “prevalence”, “pregnancy”, and “pregnant women” (See S1 Text: supplementary file for the details of the databases searches). The electronic search was enhanced through a manual appraisal of the bibliographies of identified publications and relevant reviews. When necessary, corresponding authors were contacted for additional data or information. We did not apply any geographic or language restrictions. For studies published in languages other than English, we used ‘Google Translate’. We included population-based cross-sectional and prospective cohort studies that reported the prevalence of ATI in pregnant women. We excluded editorials, commentaries, reviews, case-control studies, case series, articles focusing only on particular groups of pregnant women (e.g., HIV-positive, psychiatric patients, and women with a history of medical issues during pregnancy, with high risk pregnancies and/or with other complications), articles including non-pregnant women, articles including only pregnant women with ATI, articles reporting only IgG or IgM seropositivity rates in pregnant women, or focusing only on comparative evaluations of diagnostic methods for ATI. If articles contained overlapping data, we retained the study with the largest number of reported ATI.

After deleting duplicate studies retrieved from the different searched databases, the titles and abstracts were screened for eligibility by two investigators (H.B. and A.T.). Full texts of potentially eligible studies were screened, and any discrepancies between the two investigators were resolved by consensus with a review by a third investigator (A.R.).

The main outcome of interest in this meta-analysis was the prevalence of ATI in pregnant women. Prevalence was defined as the number of women with ATI divided by the total number of pregnant women screened. We accepted individual authors’ definitions (criteria) of ATI, including: (a) low IgG avidity (for cross-sectional studies); (b) *Toxoplasma* seropositivity for both IgG and IgM (for cross-sectional studies and cohort studies), or (c) seroconversion from a *Toxoplasma* IgG negative status to an IgG positive status (with or without positive IgM) (for prospective cohort studies).

### Data extraction

Study-level data and country-specific information was extracted independently from reference sources by two investigators (M.F. and M.N.S.) and was transferred to a Microsoft Excel spreadsheet (version 2016; Microsoft, Redmond, WA, USA). If there were inconsistencies, a third reviewer (A.R.) was consulted, and a decision was made by consensus.

From each study included, we extracted the following information: author; year of publication; country; city; study period; study design; method used to diagnose ATI in pregnant women; number of pregnant women screened; number of pregnant women diagnosed with ATI; pregnancy trimester at the time of ATI diagnosis; maternal age; *T*. *gondii* seropositivity rates in pregnant women or in the overall population (if data were unavailable for pregnant women).

For each country, we also recorded information on the corresponding Earth hemisphere and WHO-defined region for this country, using the WHO database [24]. The six WHO-defined regions considered were: the Eastern Mediterranean, Africa, Western Pacific, Americas, Southeast Asia, and Europe [24]. For each country, we also recorded information on income per capita and the human development index [HDI], using the following databases: (a) the World Bank Group [25] and (b) the United Nations Development Program [26], and information on latitude, longitude, mean relative humidity, mean temperature and precipitation rate, perusing the climate-data.org (weblink: https://en.climate-data.org/) and timeanddate.com (weblink: https://www.timeanddate.com/weather/iran/tehran/climate) databases.

### Meta-analyses

First, we calculated the prevalence of ATI (and 95% confidence interval [CI] thereof) for individual countries by synthesizing the prevalence rates of all studies from the same country by simple pooling, (as the number of events [ATIs] per study was often low) and also by random-effects-model (REM) [27]. Second, we calculated the pooled prevalence of ATI (and 95% CI) for the six WHO-defined-regions by synthesizing the ATI prevalence data across individual countries within the same WHO-defined-region by REM. Third, we calculated the overall prevalence rate of ATI (and 95% CI) across all WHO-defined-regions by synthesizing the REM data across all regions. We also conducted additional post-hoc analyses to explore the prevalence of ATI in countries where more strict criteria were used to define an ATI (seroconversion OR IgG positive/IgM positive and low IgG avidity). In this latter analysis, we excluded studies that had just IgG positive and IgM positive cases. We calculated the heterogeneity between studies (within each country), across countries (within each WHO-defined-region) and across WHO-defined-regions using the *I*^2^ statistic [28]. An *I*^2^ of 50–75%, was considered large and >75% very large heterogeneity.

We also performed *a-priori* defined sub-group and meta-regression ecological analyses to explore potential drivers of heterogeneity. We used the metareg STATA command [29]. Subgroup analyses were performed according to the following parameters: global hemisphere (northern/southern, eastern/western), WHO-defined region, type of study, method used for the diagnosis of ATI during pregnancy, pregnancy trimester at the time of ATI, maternal age, country overall seropositivity rate (in pregnant women or in the whole population), study period, country income level, country HDI, latitude, longitude, mean relative humidity, mean temperature and precipitation rate. We also compared ATI prevalence rates across different WHO regions using the χ2 test. All statistical analyses were conducted using STATA v.15 (STATA Corp., College Station, Texas, USA). Results were statistically significant if the *P* value was < 0.05.

## Results

**Fig 1:**
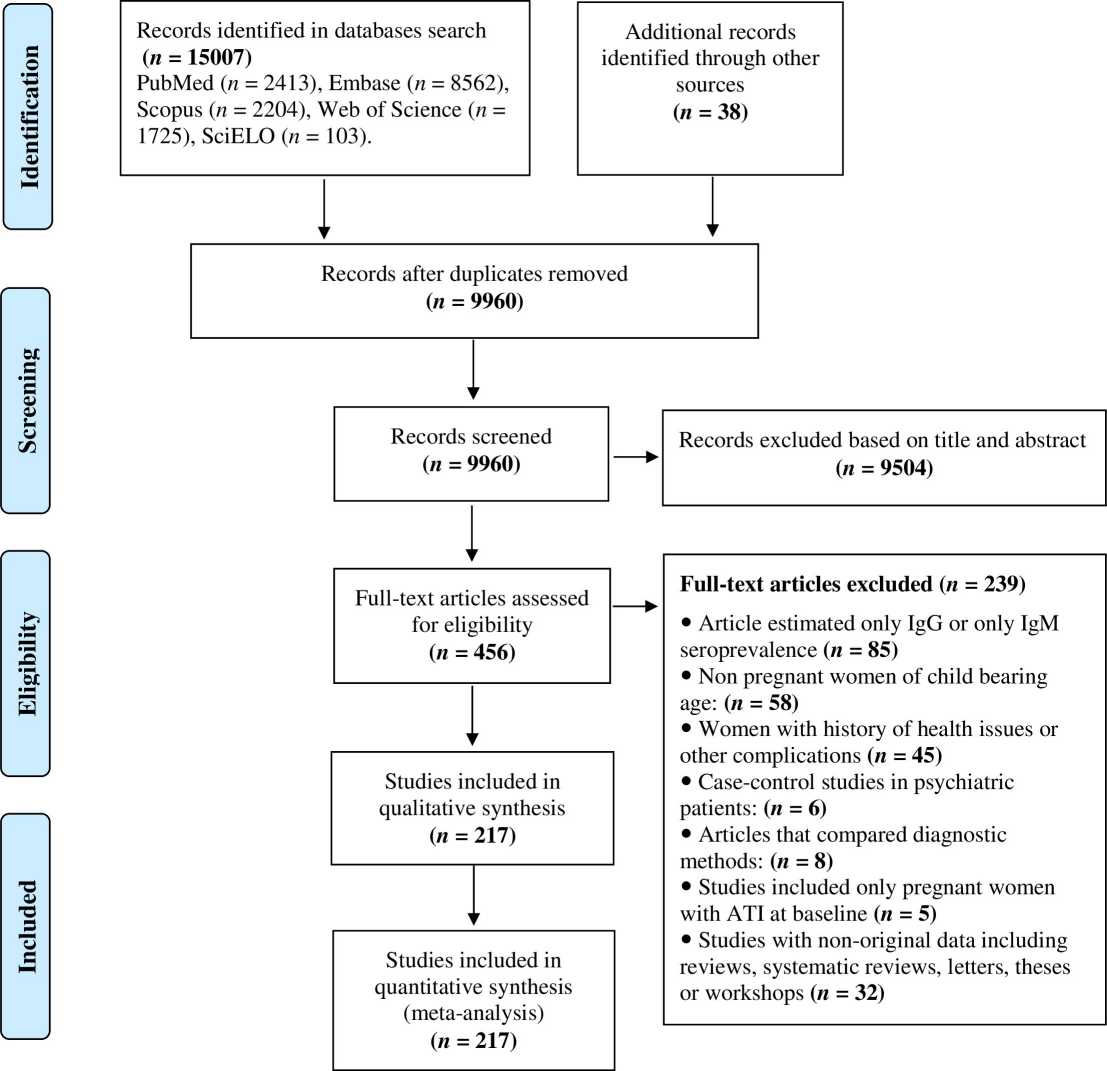
PRISMA flowchart showing the search and study selection strategy.

In total, the full texts of 456 articles were screened for eligibility, and 217 studies (220 datasets) from 74 countries were included in the meta-analysis (Fig 1). These 220 datasets represented 902,228 pregnant women and covered all six WHO-defined-regions. Most of the studies included in the meta-analysis were cross-sectional (n = 194), while 26 studies were prospective cohort studies. Most studies (n = 138) used seropositivity for both specific anti-*Toxoplasma* IgG and IgM antibodies as the criterion for the diagnosis of ATI; 47 studies employed low IgG avidity, and 35 studies used seroconversion from an IgG negative to IgG positive status (Table 1 and S1 Table).

**Table 1:** Global, regional and national pooled prevalence of acute *Toxoplasma* infection (ATI) in pregnant women (results from 217 studies [220 datasets] performed in 74 countries).

WHO regions/ country	Number of datasets	Number of pregnant women with ATI/ number of pregnant women screened	Prevalence of ATI by simple pooling % (95% CI)	Prevalence of ATI by random effect model meta-analysis % (95% CI)	Heterogeneity *I*^2^ (%)
**Global**	**220**	**6193/902228**	**0.7 (0.6–0.7)**	**1.1 (0.9–1.2)**	**97.1**
**Earth’s hemispheres (E/W)**					
Eastern	172	4892/757284	0.6 (0.6–0.7)	1.1 (0.9–1.3)	97.2
Western	48	1301/144944	0.8 (0.8–0.9)	1.0 (0.7–1.3)	96.4
**Earth’s hemispheres (S/N)**					
Southern	36	1127/143282	0.7 (0.7–0.8)	1.0 (0.6–1.3)	96.8
Northern	184	5066/758946	0.7 (0.6–0.7)	1.1 (0.9–1.3)	97.1
**Middle East and north Africa**	**53**	**671/30149**	**2.2 (2.1–2.4)**	**2.5 (1.7–3.4)**	**94.5**
Iran	22	257/9880	2.6 (2.3–2.9)	2.8 (1.5–4.5)	94.8
Saudi Arabia	13	122/11463	1.1 (0.8–1.3)	1.8 (0.8–3.1)	92.7
Egypt	5	14/1075	1.3 (0.7–2.2)	1.2 (0.0–3.4)	80.2
Yemen	3	104/1415	7.3 (6.0–8.8)	6.1 (1.6–13.3)	95.4
Sudan	3	11/744	1.4 (0.7–2.6)	1.4 (0.7–2.6)	83.7
Pakistan	1	0/180	0.0 (0.0–2.1)	0.0 (0.0–2.0)	NA
Morocco	1	5/128	3.9 (1.3–8.9)	3.9 (1.3–8.9)	NA
Lebanon	1	46/2456	1.9 (1.4–2.5)	1.9 (1.4–2.5)	NA
Kuwaiti	1	9/224	4.0 (1.9–7.5)	4.0 (1.9–7.5)	NA
United Arab Emirates	1	47/301	15.6 (11.7–20.2)	15.6 (11.7–20.2)	NA
Tunisia	1	53/2070	2.6 (1.9–3.3)	2.6 (1.9–3.3)	NA
Israel	1	3/213	1.4 (0.2–4.0)	1.4 (0.3–4.1)	NA
**African region**	**27**	**257/8990**	**2.8 (2.5–3.2)**	**1.6 (0.7–2.9)**	**93.5**
Ethiopia	3	14/665	2.1 (1.1–3.5)	2.0 (0.8–3.6)	35.0
Burkina Faso	3	7/834	0.8 (0.3–1.7)	0.5 (0.0–3.2)	88.6
Ghana	3	13/452	2.8 (1.5–4.9)	1.3 (0.0–8.2)	92.4
Senegal	3	108/1464	7.3 (6.1–8.8)	5.0 (0.0–18.7)	98.2
Nigeria	2	29/734	3.9 (2.7–5.6)	3.2 (2.0–4.6)	94.3
Tanzania	2	23/604	3.8 (2.4–5.6)	1.9 (0.9–3.2)	67.5
Algeria	2	20/1171	1.7 (1.0–2.6)	1.4 (0.7–2.1)	99.7
Benin	2	0/494	0.0 (0.0–0.7)	0.0 (0.0–0.7)	NA
Gabon	1	22/839	2.6 (1.7–3.9)	2.6 (1.7–3.9)	NA
Rwanda	1	15/384	3.9 (2.2–6.3)	3.9 (2.2–6.4)	NA
Zambia	1	0/411	0.0 (0.0–0.8)	0.0 (0.0–0.9)	NA
Angola	1	0/300	0.0 (0.0–1.2)	0.0 (0.0–1.2)	NA
Congo	1	2/378	0.5 (0.0–1.8)	0.5 (0.0–1.8)	NA
Cameroon	1	3/110	2.7 (0.5–7.7)	2.7 (0.5–7.7)	NA
Mozambique	1	1/150	0.6 (0.0–3.6)	0.6 (0.0–3.6))	NA
**Western Pacific Region**	**23**	**505/46288**	**1.0 (0.9–1.2)**	**1.0 (0.5–1.6)**	**96.6**
China	11	451/24245	1.8 (1.6–2.0)	1.5 (0.9–2.2)	93.4
Taiwan	4	23/1233	1.9 (1.2–2.9)	1.7 (0.9–2.8)	30.4
Malaysia	2	1/500	0.2 (0.0–1.1)	0.1 (0.0–0.8)	99.8
Australia	2	14/10816	0.1 (0.0–0.2)	0.1 (0.0–0.2)	76.8
New Zealand	1	12/500	2.4 (1.2–4.2)	2.4 (1.2–4.2)	NA
Japan	1	3/2969	0.1 (0.0–0.2)	0.1 (0.0–0.3)	NA
Vietnam	1	0/300	0.0 (0.0–1.2)	0.0 (0.0–0.2)	NA
South Korea	1	1/5725	0.01 (0.0–0.01)	0.0 (0.0–0.1)	NA
**Latin America & Caribbean region**	**33**	**1140/129358**	**0.9 (0.8–1.0)**	**1.0 (0.7–1.4)**	**96.5**
Brazil	23	916/109543	0.8 (0.7–0.9)	1.0 (0.6–1.5)	97.0
Mexico	3	1/1120	0.1 (0.0–0.4)	0.0 (0.0–0.3)	0
Colombia	2	42/1892	2.2 (1.6–3.0)	2.2 (1.6–2.9)	99.8
Venezuela	1	10/678	1.4 (0.7–2.7)	1.5 (0.7–2.7)	NA
Caribbean islands	1	0/437	0.0 (0.0–0.8)	0.0 (0.0–0.8)	NA
Trinidad and Tobago	1	34/450	7.6 (5.2–10.3)	7.6 (5.3–10.4)	NA
Argentina	1	121/13632	0.9 (0.7–1.0)	0.9 (0.7–1.1)	NA
Cuba	1	16/1606	0.9 (0.6–1.6)	1.0 (0.6–1.6)	NA
**South-East Asian Region**	**18**	**55/6391**	**0.8 (0.6–1.1)**	**0.8 (0.3–1.5)**	**82.2**
India	6	27/1879	1.4 (0.9–2.1)	1.5 (0.5–3.0)	75.1
Thailand	7	89/3230	0.5 (0.3–1.0)	0.5 (0.0–1.5)	86.3
Sri Lanka	2	2/829	0.2 (0.0–0.8)	0.2 (0.0–0.7)	98.9
Bangladesh	2	7/238	2.9 (1.2–5.9)	2.8 (0.9–5.4)	95.0
Myanmar	1	0/215	0.0 (0.0–1.7)	0.0 (0.0–1.7)	NA
**Europe region**	**66**	**3565/681052**	**0.5 (0.5–0.6)**	**0.5 (0.4–0.7)**	**98.2**
Turkey	17	248/74907	0.3 (0.2–0.4)	0.4 (0.2–0.7)	95.9
Italy	7	340/61846	0.5 (0.4–0.6)	0.5 (0.4–0.6)	98.3
Spain	5	62/26475	0.2 (0.1–0.3)	0.3 (0.1–0.8)	94.6
Poland	3	79/15213	0.5 (0.4–0.6)	0.5 (0.3–0.7)	51.5
United Kingdom	3	39/16846	0.2 (0.1–0.3)	0.2 (0.1–0.3)	52.1
Austria	3	965/167032	0.5 (0.5–0.6)	1.2 (0.3–2.7)	99.6
Sweden	3	28/48423	0.05 (0.49–0.5)	0.1 (0.0–0.3)	91.6
Slovenia	3	354/82304	0.4 (0.3–0.6)	0.4 (0.3–0.6)	91.2
Germany	2	28/9757	0.2 (0.1–0.4)	0.3 (0.2–0.4)	99.8
Netherland	2	58/28549	0.2 (0.1–0.3)	0.2 (0.1–0.2)	98.6
Belgium	2	286/27450	1.0 (0.9–1.1)	1.0 (0.9–1.1)	99.7
Serbia	2	22/3440	0.6 (0.4–0.9)	0.6 (0.4–0.9)	99.7
Portugal	1	0/155	0.0 (0.0–2.0)	0.0 (0.0–2.4)	NA
Kosovo	1	4/334	1.1 (0.3–3.0)	1.2 (0.3–3.0)	NA
Albania	1	2/496	0.4 (0.4–1.4)	0.4 (0.0–1.4)	NA
Greece	1	185/5532	3.3 (2.8–3.8)	3.3 (2.9–3.9)	NA
Denmark	1	35/5402	0.6 (0.4–0.9)	0.6 (0.4–0.9)	NA
Hungary	1	78/17735	0.4 (0.3–0.6)	0.4 (0.3–0.5)	NA
Russia	1	393/9365	4.1 (3.7–4.6)	4.2 (3.8–4.6)	NA
Czech Republic	1	20/1409	1.4 (0.8–2.2)	1.4 (0.9–2.2)	NA
France	1	35/2216	1.6 (1.1–2.2)	1.6 (1.1–2.2)	NA
Norway	1	47/32033	0.1 (0.1–0.2)	0.1 (0.1–0.2)	NA
Finland	1	25/16733	0.1 (0.0–0.2)	0.1 (0.1–0.2)	NA
Scotland	1	10/4548	0.2 (0.1–0.4)	1.1 (0.9–1.2)	NA
Cyprus	1	107/17631	0.6 (0.5–0.7)	0.6 (0.4–0.9)	NA
Switzerland	1	115/5221	2.0 (1.8–2.6)	2.2 (1.8–2.6)	NA

Abbreviations: NA: not applicable

WHO regions are sorted according to prevalence rates

Countries are sorted according to number of studies included

**Fig 2:**
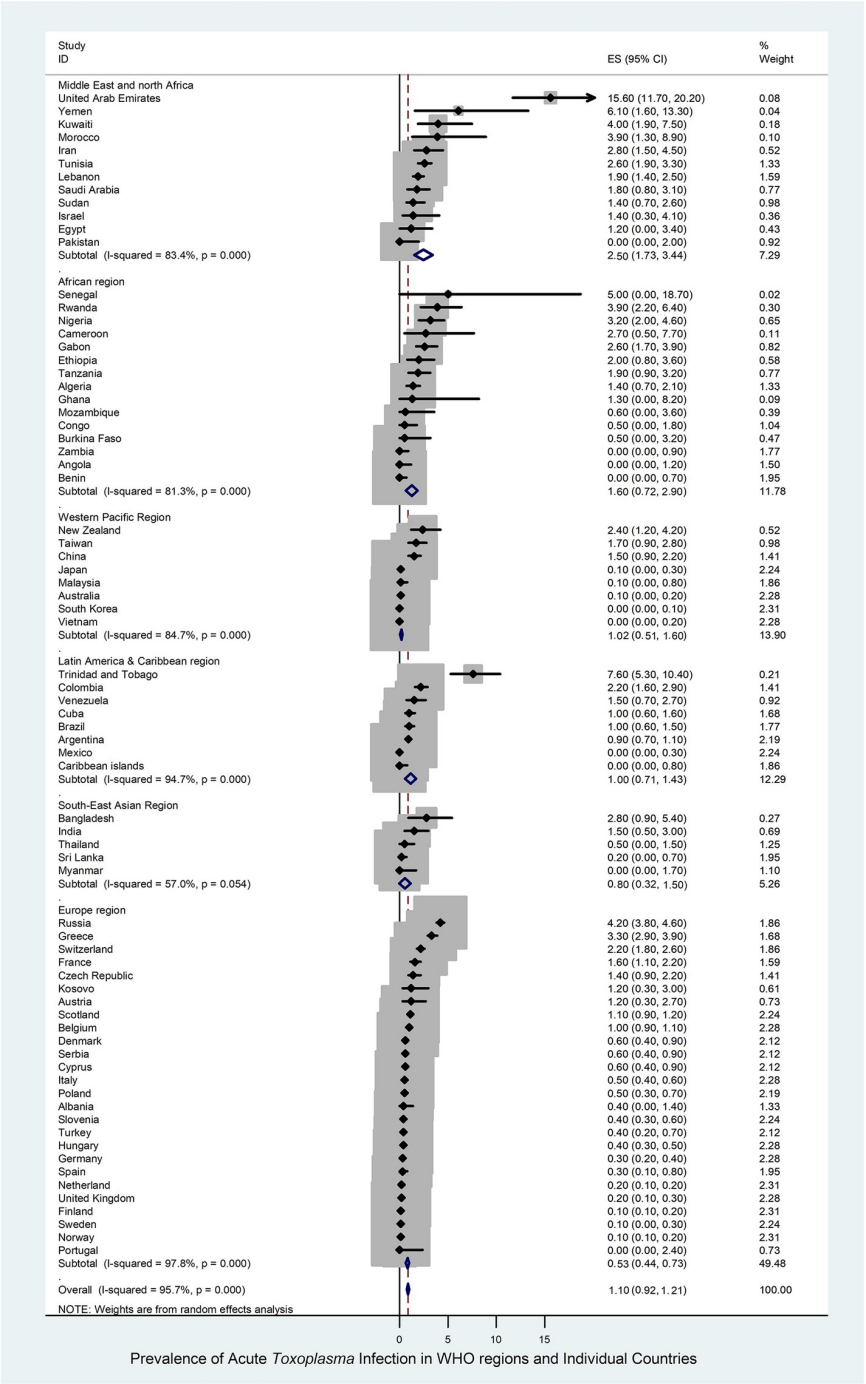
Forest Plot of the prevalence of acute *Toxoplasma* infection (ATI) by WHO-region and globally. ES: estimated prevalence of ATI for WHO regions and individual countries.

**Fig 3:**
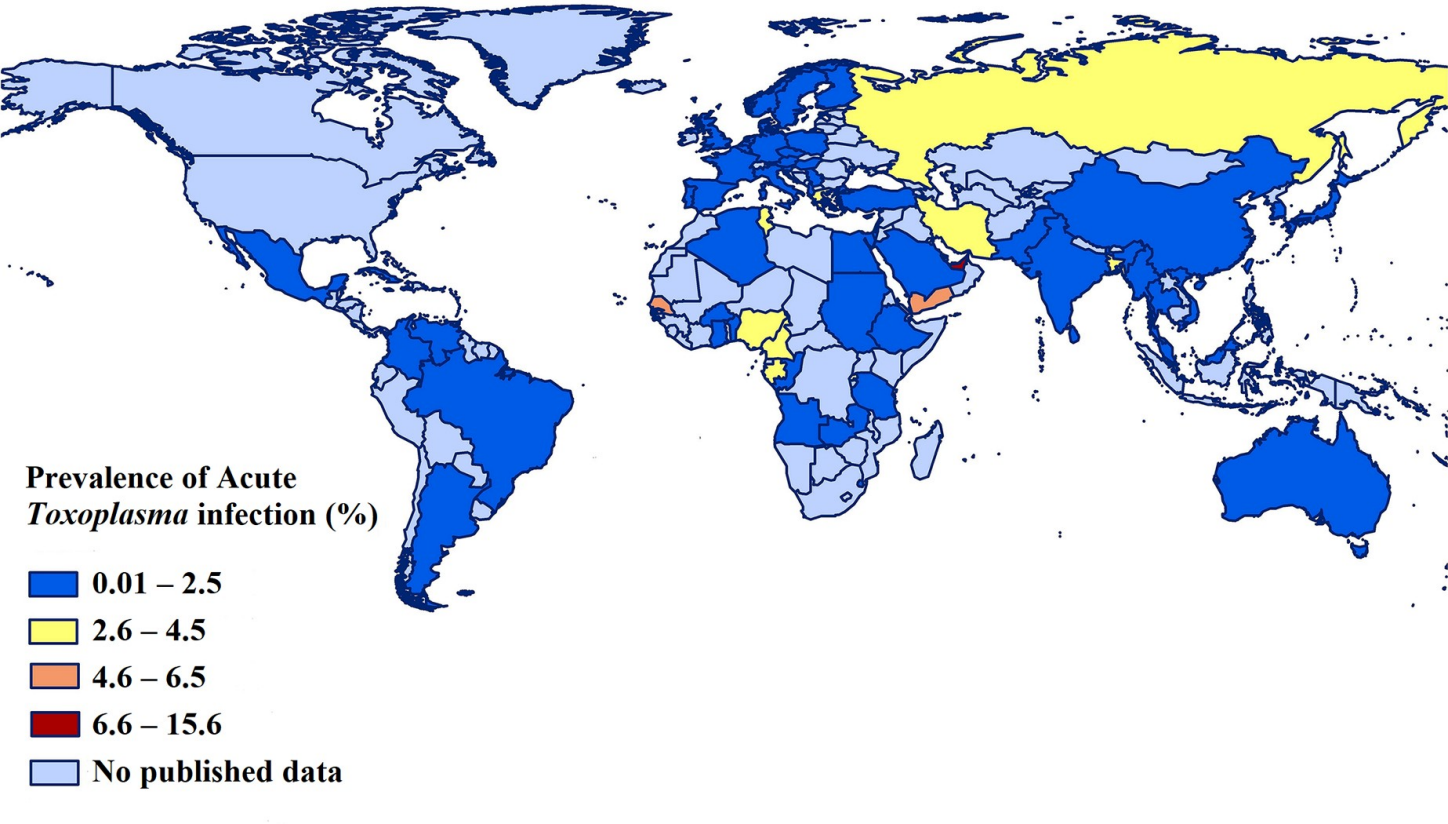
Prevalence of acute *Toxoplasma* infection (ATI) in pregnant women in different countries using geographic information system (GIS). We used ArcGIS program (version 10.2.2; ESRI, Redlands, California, USA) to draw this figure.

The results were similar using simple pooling and REM analyses. Thus, we show here only REM analysis results. Results by simple pooling are shown in Tables 1–3. The global prevalence of ATI in pregnant women across 74 countries was 1.1% (95% CI: 0.9–1.2%) (6,193/902,228) (Table 1). There was large heterogeneity in prevalence estimates among countries (*I*^2^ = 97.1%) (Table 1, Fig 2). Prevalence estimates in different hemispheres, WHO-defined regions and individual countries are shown in Table 1 and Figs 2 and 3. The overall ATI prevalence rates in the northern and southern hemispheres were similar (1.1%; 95% CI: 0.9–1.3 and 1.0%; 95% CI: 0.6–1.3, respectively), as they were in the eastern and western hemispheres (1.1%; 95% CI: 0.9–1.3 and 1.0%; 95% CI: 0.7–1.3, respectively) (S1 Fig, S2 Fig and Table 1).

**Table 2:** Prevalence of acute *Toxoplasma* infection (ATI) in pregnant women according to *a priori* defined subgroups.

Parameters/ subgroups	Number of datasets	Number of pregnant women with ATI/ number of pregnant women screened	Prevalence of ATI by simple pooling % (95% CI)	Prevalence of ATI by random effect model meta-analysis % (95% CI)	Heterogeneity *I*^2^ (%)	Univariate analyses *(p value by Chi2 test)*
**Type of study design**						< 0.001
Cross-sectional	194	4871/694955	0.7 (0.6–0.7)	1.0(0.9–1.2)	96.5	
Longitudinal cohort	26	1322/207273	0.6 (0.6–0.7)	1.1(0.9–1.2)	98.8	
**Type of ATI diagnosis method**						< 0.001
Positive for both IgG and IgM	138	2158/168419	1.3 (1.2–1.3)	1.5 (1.3–1.8)	94.4	
Low IgG avidity	47	1409/275630	0.5 (0.4–0.5)	0.5 (0.3–0.7)	97.4	
Seroconversion	35	2626/458179	0.6 (0.5–0.6)	0.7 (0.5–0.9)	98.7	
**Pregnancy trimester⁂**						< 0.001
First trimester	15	193/14153	1.3 (1.2–1.6)	1.7 (0.7–3.0)	92.1	
Second trimester	14	58/9584	0.6 (0.4–0.8)	1.0 (0.3–1.9)	84.8	
Third trimester	14	41/4366	0.9 (0.6–1.3)	0.1 (0.0–1.1)	83.7	
**Maternal age (years)⁂**						< 0.001
<20	14	84/4594	1.8 (1.5–2.3)	2.6 (1.0–4.8)	88.9	
20–30	14	215/34296	0.6 (0.5–0.7)	2.2 (1.1–3.5)	96.9	
>30	14	97/17811	0.5 (0.4–0.6)	1.6 (0.6–3.0)	91.5	
Seropositivity rate* (%)						< 0.001
0–10	23	342/51409	0.6 (0.6–0.7)	0.5 (0.2–0.8)	93.6	
10–20	28	834/143128	0.6 (0.5–0.6)	0.7 (0.3–1.1)	98.3	
20–30	41	494/81074	0.6 (0.5–0.7)	1.2 (0.7–1.7)	96.4	
30–40	51	2008/334653	0.6 (0.5–0.6)	1.1 (0.9–1.4)	97.3	
40–50	32	1004/122251	0.8 (0.7–0.9)	1.4 (1.0–1.9)	97.0	
>50	45	1511/169713	0.9 (0.8–0.9)	1.1 (0.9–1.2)	96.2	
**Sample size**						< 0.001
<1000	137	1180/51414	2.2 (2.1–2.4)	1.6 (1.2–2.0)	92.2	
1000–5000	47	1034/121935	0.8 (0.8–0.9)	0.7 (0.5–1.0)	96.4	
5000–10000	13	1165/89785	1.3 (1.2–1.4)	0.9 (0.4–1.7)	99.1	
10000–20000	10	822/144194	0.5 (0.5–0.6)	0.5 (0.2–0.8)	98.3	
>20000	13	1992/494900	0.4 (0.3–0.4)	0.3 (0.2–0.5)	99.2	
**Study year**						< 0.001
1988–1999	37	1236/222258	0.6 (0.5–0.6)	0.8 (0.5–1.0)	97.1	
2000–2004	19	686/105799	0.6 (0.6–0.7)	1.2 (0.6–1.9)	98.6	
2005–2009	35	1506/157084	0.9 (0.9–1.0)	1.1 (0.8–1.6)	97.5	
2010–2014	65	1066/172706	0.6 (0.6–0.7)	1.2 (0.9–1.5)	96.4	
2015–2018	64	1699/244381	0.7 (0.6–0.8)	1.1 (0.9–1.2)	95.8	

* Overall *T*. *gondii* seropositivity rate (latent *Toxoplasma* infection) in pregnant women or overall population seropositivity rate, as reported in the individual studies

⁂ According to pregnancy trimester and maternal age, data for ATI were reported only in small numbers of studies (15 and 14, respectively). In the remaining studies, no data were reported.

**Table 3:** Prevalence of acute *Toxoplasma* infection (ATI) in pregnant women based on subgroups according to different socio-demographic and geographic parameters.

Parameters/subgroups	Number of datasets	Number of pregnant women with ATI/ number of pregnant women screened	Prevalence of ATI by simple pooling % (95% CI)	Prevalence of ATI by random effect model meta-analysis % (95% CI)	Heterogeneity *I*^2^ (%)	Univariate analyses (*Chi2*)
**Income**						< 0.001
Low	16	170/4973	3.4 (2.9–3.9)	1.6 (0.4–3.7)	94.8	
Lower middle	33	269/10964	2.4 (2.2–2.7)	1.5 (0.8–2.5)	90.5	
Upper middle	10	2203/257965	0.8 (0.8–0.9)	1.1 (0.9–1.4)	96.2	
**High**	71	3551/628326	0.5 (0.5–0.6)	0.8 (0.6–1.0)	98.1	
**Human development index (HDI)**						< 0.001
Low	23	312/7488	4.1 (3.7–4.6)	2.3 (1.0–4.1)	94.8	
Medium	23	71/5486	1.3 (1.0–1.6)	1.1 (0.5–1.8)	80.4	
High	105	2188/241036	0.9 (0.8–0.9)	1.2 (0.9–1.4)	96.3	
Very high	69	3622/648218	0.5 (0.5–0.6)	0.7 (0.5–0.9)	98.2	
**Latitude**						< 0.001
0–10°	26	159/10370	1.5 (1.3–1.8)	1.0 (0.4–1.7)	88.6	
10–20°	37	524/26339	2.0 (1.8–2.2)	1.8 (1.0–2.8)	95.5	
20–30°	42	886/109931	0.8 (0.7–0.9)	1.1 (0.8–1.5)	95.1	
30–40°	63	1542/168558	0.9 (0.8–1.0)	1.4 (1.0–1.8)	97.3	
40–50°	32	2054/372711	0.5 (0.5–0.6)	0.7 (0.5–0.9)	97.8	
≥50°	20	1028/214319	0.4 (0.4–0.5)	0.4 (0.2–0.7)	98.7	
**Longitude**						< 0.001
0–10°	32	733/125213	0.5 (0.5–0.6)	0.6 (03–0.9)	95.8	
10–20°	31	2037/419798	0.5 (0.5–0.6)	0.8 (0.5–1.0)	98.4	
20–30°	15	365/62723	0.6 (0.5–0.6)	0.5 (0.2–0.9)	97.2	
30–40°	33	468/54138	0.8 (0.7–0.9)	1.1 (0.7–1.5)	96.6	
40–50°	35	910/101188	0.9 (0.8–1.0)	2.2 (1.5–2.9)	97.8	
50–60°	22	624/70761	0.9 (0.8–1.0)	2.0 (1.3–2.9)	96.6	
60–70°	2	44/1128	3.9 (2.8–5.2)	3.4 (2.4–4.5)	99.8	
70–80°	10	451/13711	3.2 (2.9–3.6)	1.0 (0.2–2.3)	94.6	
80–90°	4	29/2477	1.1 (0.7–1.8)	1.3 (0.3–2.8)	76.9	
90–100°	3	7/453	1.5 (0.6–3.2)	1.1 (0.0–5.0)	81.7	
100–110°	15	81/7081	1.1 (0.9–1.4)	0.5 (0.1–1.2)	90.2	
110–120°	5	281/2503	1.1 (1.0–1.2)	0.9 (0.1–2.4)	98.8	
≥ 120°	13	163/18554	0.9 (0.7–1)	1.0 (0.4–1.9)	94.8	
**Relative humidity (%)**						< 0.001
<40	13	202/11982	1.7 (1.5–1.9)	2.2 (0.8–4.3)	96.7	
40–50	15	120/14601	0.8 (0.6–1.0)	1.3 (0.7–2.1)	86.8	
50–60	29	418/42036	0.9 (0.9–1.0)	1.6 (1.0–2.2)	94.1	
60–70	52	1237/147466	0.8 (0.8–0.9)	0.8 (0.5–1.2)	97.2	
70–80	86	2923/645684	0.4 (0.4–0.5)	1.1 (0.9–1.4)	97.7	
≥80	25	1293/240459	0.5 (0.5–0.6)	0.5 (0.3–0.8)	97.1	
**Mean temperature (⁰C)**						< 0.001
≤7	11	574/143487	0.4 (0.3–0.4)	0.4 (0.1–0.9)	99.2	
8–14	59	251287/465639	0.5 (0.5–0.6)	0.9 (0.7–1.1)	97.1	
15–20	60	1970/210907	0.9 (0.9–1.0)	1.2 (0.9–1.5)	97.1	
20–30	90	1062/82195	1.2 (1.2–1.4)	1.3 (0.9–1.6)	94.1	
**Precipitation (mm)**						< 0.001
0–250	24	195/14715	1.3 (1.1–1.5)	1.7 (0.9–2.8)	93.2	
250–500	37	1231/91765	1.3 (1.2–1.4)	2.0 (1.3–2.9)	98.3	
500–1000	84	2925/545367	0.5 (0.5–0.6)	0.8 (0.6–1.0)	97.4	
1000–2000	65	1548/227985	0.6 (0.6–0.7)	0.9 (0.7–1.2)	95.0	
≥2000	10	294/22396	1.3 (1.2–1.4)	1.1 (0.9–1.2)	92.3	

With regard to the WHO-defined-regions, the highest prevalence of ATI in pregnant women (2.5%; 95% CI: 1.7–3.4%; 671/30,149) was reported for the Eastern Mediterranean region, and the lowest prevalence (0.5%; 95% CI: 0.4–0.7%; 3,568/681,265) was in the European region (Table 1). The prevalence estimates of ATI in pregnant women in other WHO-defined regions were: 1.6% (95% CI: 0.7–2.9%) in Africa, 1.0% (95% CI: 0.5–1.6%) in the Western Pacific, 1.0% (95% CI: 0.7–1.4%) in Latin America and the Caribbean, and 0.8% (95% CI: 0.3–1.5%) in South-East Asia. Fig 3 shows a geographic information system (GIS) map with the prevalence rates of ATI in pregnant women in different countries.

With regard to the post-hoc exploratory analyses based on studies that used strict criteria to define ATI, we identified 87 such studies from 47 countries. The overall prevalence of ATI in pregnant women across these 47 countries was 0.6% (95% CI: 0.4–0.7%; 4,035/733,809). According to this analysis, the prevalence estimates of ATI in pregnant women in WHO-defined-regions were (from highest to lowest): 2.2% (95% CI: 0.2–6.1%) in Africa, 1.9% (95% CI: 1.0–3.0%) in the Eastern Mediterranean region, 0.6% (95% CI: 0.1–3.2%) in the Western Pacific, 0.5% (95% CI: 0.2–0.9%) in the Latin American and Caribbean region, 0.5% (95% CI: 0.1–1.3%) in South-East Asia and 0.4% (95% CI: 0.3–0.6%) in the European region (S2 Table).

In subgroup analyses, according to study design, the global prevalence of ATI in prospective cohort studies was 1.1% (95% CI: 0.9–1.2%) and was similar to that in cross-sectional studies (1.0%; 95% CI: 0.9–1.2%) (Table 2). With respect to the type of diagnostic method used, the prevalence rates according to the diagnostic criteria used to define ATI were: 1.5% (95% CI: 1.3–1.8%), using seropositivity for both IgG and IgM, 0.5% (95% CI: 3–0.7%) using low IgG-avidity (along with positive IgG and IgM), and 0.7% (95% CI: 0.5–0.9%) using seroconversion from an IgG negative to an IgG positive status (Table 2). In subgroup analysis, according to pregnancy trimesters, only 15 studies contained relevant data; the pooled prevalence rates for ATI were 1.7% (95% CI: 0.7–1.3%), 1.0% (95% CI: 0.3–1.9%) and 0.1% (95% CI: 0.0–1.1%) for ATI in the first, second, and third trimesters, respectively (Table 2). Fourteen studies reported the prevalence of ATI in different age groups; the pooled prevalence rates of ATI in pregnant women with ages of <20, 21–30, and >31 years were 2.6% (95% CI: 1.0–4.8%), 2.2% (95% CI: 1.1–3.5%) and 1.6% (95% CI: 0.6–3%), respectively (Table 2). The results of additional subgroup analyses, according to study sample size and study year, are shown in Table 2.

In subgroup analyses, with respect to sociodemographic parameters, countries with high income levels and higher HDI had a significantly lower prevalence of ATI (*P*-value <0.001). In subgroup analysis, according to income level, the highest and lowest prevalence of ATI were found in low (1.6%; 95% CI: 0.4–3.7%) and high (0.8%; 95% CI: 0.6–1%) income countries, respectively. According to HDI, the highest prevalence of ATI were found in countries with low HDIs (2.3%; 95% CI: 1–4.1%), and the lowest prevalence in countries with high HDIs (0.7%; 95% CI: 0.5–0.9%) (Table 3). Additional subgroup analyses, according to geographic and climate parameters (latitude, longitude, humidity, mean temperature, and precipitation), are shown in Table 3.

**Fig 4:**
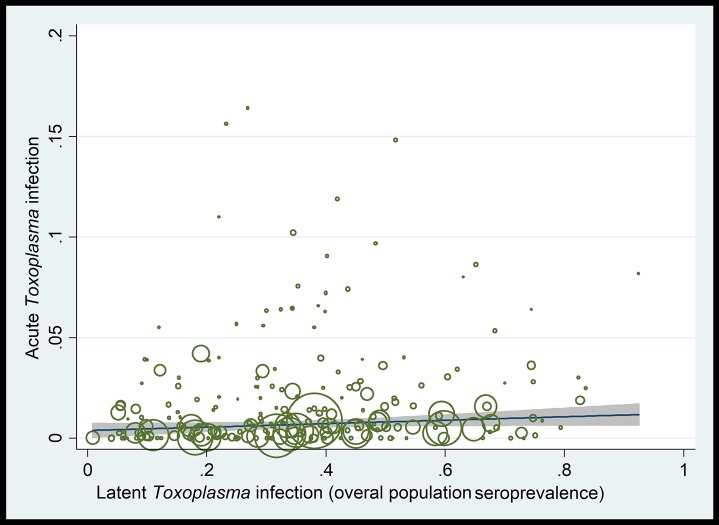
Meta-regression analysis showing a non-significant ecological upward trend in the rates of acute *Toxoplasma* infection (ATI) with increasing overall population *T*. *gondii* seroprevalence rates (latent *Toxoplasma* infection) in pregnant women.

Meta-regression analyses showed a non-significant increasing trend in ATI prevalence with increasing overall seropositivity rates (latent *Toxoplasma* infection) in pregnant women or in the overall population (coefficient [*C*] = 0.008; *P*-value = 0.17) (Fig 4, Table 2). There was a non-significant decreasing trend in ATI prevalence with larger study-sample sizes (*C* = -5. 79e-08; *P*-value = 0.1) and over time (*C* = 0.0007; *P*-value = 0.57). There was a significant decreasing trend with increasing income levels (*C* = -0.005; *P*-value = 0.03) and HDI (*C* = -0.005; *P*-value = 0.006) (S3 Fig, Table 2). A significant decreasing trend in ATI prevalence was also seen with increasing geographical latitude (*C* = -0.00026, *P*-value = 0.005); while an overall significant increasing trend was seen with increasing longitude (*C* = 0.0008, *P*-value = 0.02), although prevalence increased between longitudes 0–70° and then decreased between longitudes 70–130° (S3 Fig, Table 3). Furthermore, a significant increasing trend in ATI prevalence was seen with increasing mean environmental temperature (*C* = 0.0004; *P*-value = 0.02); while a non-significant decreasing trend was seen with increasing relative humidity (*C* = -0.0001, *P*-value = 0.18) and increasing annual precipitation (*C* = -6.41, *P*-value = 0.81) (S3 Fig).

## Discussion

To our knowledge, this is the first study to evaluate published evidence on the global and regional prevalence of ATI in pregnant women. We estimated that, globally, approximately 1.1% of pregnant women are acutely infected with *T*. *gondii* during gestation. Following more strict criteria for the definition of ATI, we have found that the prevalence of ATI in pregnant women was about 0.6%. These prevalence rates represent a significant burden of infection in pregnant women, and suggest that a large number of newborns are at risk of acquiring congenital toxoplasmosis in utero and at risk of developing long-term sequelae from the disease, if left undiagnosed and untreated. Our findings support the need for universal screening of pregnant women during the gestation period. Approximately 50% of pregnant women with ATI are asymptomatic or may have mild non-specific flu-like symptoms; without universal screening throughout gestation (ideally every 1–3 months) these cases will remain undiagnosed. Prompt diagnosis and treatment of ATI during gestation, and ideally within 3–4 weeks from the time of acute maternal infection, could be helpful to prevent MTCT of congenital toxoplasmosis and ameliorate the severity of the disease [7, 10, 11, 17, 30, 31]. The spectrum of disease resulting from congenital toxoplasmosis in countries without universal prenatal screening for *Toxoplasma* infection is significantly more severe as compared with countries where such prenatal screening and treatment programs are routinely implemented. ATI, identified during pregnancy, allows for promptly initiated ante-natal and post-natal treatment for congenital toxoplasmosis [4, 32, 33].

In our analyses, the overall prevalence of ATI varied across regions, with the lowest prevalence in European countries and the highest prevalence in Eastern Mediterranean and African countries. These results are in agreement with the estimated global incidence of congenital toxoplasmosis from a WHO-supported study [3] that showed the highest incidence of congenital toxoplasmosis in the Middle East and in some low-income African countries. Using an estimated ATI prevalence of 0.6% in pregnant women, based on strict criteria to define ATI, and ~140 million births per year globally (2017), it could be projected that ~ 840,000 babies per year are born annually to women with ATI. With an overall mother-to-child transmission risk of ~24% [14], ~ 201,600 children annually could be born with congenital toxoplasmosis (14.4 CT cases per 10,000 live births worldwide). These estimates are consistent with estimates by Torgesson and Mastroiacovo [3]. Using a number of 131 million births in 2008, these authors estimated that the global incidence of congenital toxoplasmosis is 190,000 (95% CI: 179,300–206,300) cases per year (equating to 15 CT cases per 10,000 live births worldwide) [3].

Very few (n = 15) studies reported information on the gestational age of the pregnant women at the time of ATI. The prevalence of ATI in the first trimester (1.7%) was significantly higher than the prevalence in the second (1.0%) and third (0.1%) trimesters. It is well known that gestational age at the time of maternal infection has an impact on the risk of vertical transmission and the risk of developing severe, symptomatic disease in the fetus and the newborn [1, 5, 12]. The risk of mother-to-child transmission increases geometrically with advancing gestational age at the time of ATI, while the risk for symptomatic disease in the fetus or newborn follows a relatively reverse trajectory [8]. Nevertheless, recent data show that severe congenital toxoplasmosis can occur even with late gestation ATI. High risk for ATI early in gestation could have important public health implications. Many *T*. *gondii* seronegative pregnant women may be unaware about their pregnancy status in the early weeks of gestation, and might be involved in activities that expose them to *T*. *gondii* (e.g., through the ingestion of raw or under-cooked meat, unwashed vegetables, or untreated or inappropriately treated water, through environmental exposure to soil or through contact with cats). Therefore, educating people about measures to prevent *Toxoplasma* infection in women when or before they are planning to become pregnant, and implementing universal prenatal screening programs during gestation are key preventative measures.

A significantly higher prevalence of ATI was reported in younger, compared with older, pregnant women. The ATI prevalence rates were 2.6% in women of < 20 years of age compared with 1.6% in women > 31 years of age. Although, in general, the seroprevalence of *T*. *gondii* in a population increases with advancing age [34–36], the relatively contradictory results for ATI prevalence rates in our meta-analysis could be explained by the fact that older pregnant women are likely to have been exposed to *T gondii* earlier in life, and, thus, are no longer at risk.

Although not statistically significant, there was an upward trend in the prevalence of ATI in pregnant women with increasing *T*. *gondii* seropositivity rates in the general population. If the *T*. *gondii* seroprevalence in a country or a population is high, then a small number of pregnant women would be expected to be at risk of acquiring ATI during gestation, because most women would be seropositive, as a result of (usually asymptomatic) infection, prior to conception. This proposal is consistent with results for Latin America, for example. Nevertheless, a high seroprevalence would also mean that there is higher risk of *T*. *gondii* exposure in seronegative individuals via environmental or occupational sources or through *T*. *gondii* contaminated food or water [3].

Higher prevalence rates of ATI were reported in countries with lower income and HDI levels, lower latitude and longitude, lower humidity, and higher mean annual temperatures. It is well known that the optimum environmental conditions required for the survival of *Toxoplasma* oocysts in the environment include a warm and humid climate [37, 38]. Moreover, the prevalence of human toxoplasmosis is higher in areas with a hot and humid climate. However, we found relatively conflicting results for the association of ATI prevalence with the humidity and precipitation parameters for individual countries. The highest prevalence of ATI was seen in the Middle East and North Africa, where the humidity and precipitation rates are lower than in other regions. Our interpretation is that socio-demographic variables (income and HDI levels) as well as cultural and culinary habits have more powerful effects on the prevalence of ATI as compared with environmental variables. Pregnant women living in countries with high income and HDI levels likely have better access to health care and might have more knowledge about the risks of infectious diseases (including ATI) during pregnancy. Furthermore, regular maternal screening and a greater awareness of health professionals about the risks of ATI in developed countries could be effective in reducing the incidence of exposure during pregnancy [39]. Cultural or culinary habits, particularly the consumption of semi-cooked or raw meat (including lamb), are important factors in the acquisition of *Toxoplasma* infection [35]. This might explain the high prevalence of ATI in pregnant women living in the Middle East region, since Kebab, Shishlik and other recipes with relatively undercooked meats are very popular in the daily diet of people in these countries [35]. Furthermore, poor environmental hygiene, accompanied with limited awareness about the transmission routes and clinical complications of toxoplasmosis could be responsible for the high prevalence of ATI in pregnant women in the Middle East.

The strength of the present study was the large sample size (902,228 pregnant women), which allowed the regional and global prevalence rates of ATI in pregnant women to be estimated. However, some limitations need to be acknowledged. First, although we undertook a comprehensive systematic literature search of relevant databases and included a substantial amount of information and data in the meta-analysis, we limited our analyses to data published in the peer reviewed literature. Unpublished data, reported, for example, in meeting-proceedings, were not included in the present meta-analysis. Second, for some countries, only single reports were available and for several regions, including North America, Eastern Europe and Central Asia, we found no published data. Third, the methods used for the diagnosis of ATI in the different studies varied considerably, and the diagnostic sensitivity and specificity of these methods differed as well, likely resulting in some imprecision in prevalence estimates. In our meta-analysis, studies diagnosing ATI based on the presence of positive anti-*Toxoplasma* IgG and IgM serum antibodies reported statistically-significantly higher ATI prevalence rates (1.5%) than studies using low IgG avidity (along with positive IgG and IgM) or seroconversion from IgG negative to IgG positive (ATI prevalence rates of 0.5% and 0.7%, respectively). Fourth, there was a high degree of heterogeneity in the estimated ATI prevalence rates by country. This is expected in global estimates across time periods and locations. In our meta-regression analyses, we tried to explore reasons for this heterogeneity and identified several socioeconomic, geographic and climate parameters that might explain heterogeneity, although, in most cases, the identified trends were not statistically significant. Finally, all of our sub-group and meta-regression analyses, were ecological analyses and several of the statistically significant subgroup differences identified might also be explained by unaccounted confounders.

In conclusion, we estimated a prevalence rate of ATI in pregnant women, both regionally and globally, which poses a serious public health risk, and suggests that a significant number of fetuses and newborns are at risk of MTCT transmission of congenital toxoplasmosis. The Sustainable Development Goal of the United Nations calls for a radical reduction of newborn, child and maternal mortality, focused on ending preventable deaths before 2030. Our data provide useful information for public health policymakers, nationally or internationally, to prioritize prevention efforts and intervention programs that will improve maternal and newborn health and minimize adverse outcomes due to congenital toxoplasmosis. Furthermore, our study suggests the need for a global effort to educate women of a child-bearing age about the transmission routes of toxoplasmosis and adverse effects of ATI on the developing fetus and newborn, as well as measures to avoid such exposure risks. Congenital toxoplasmosis is clearly a preventable disease. Universal prenatal screening programs for *Toxoplasma* infection throughout gestation should be implemented in all countries. Screening programs using novel, well-validated diagnostic platforms, like point-of-care tests for *Toxoplasma* infection [40–42], could mitigate the cost and the implementation limitations associated with the need for large-scale screening programs for pregnant women during gestation.

## Supporting information

S1 Checklist PRISMA checklist. (DOCX)

S1 Table Main characteristics of all eligible studies reporting prevalence of acute *Toxoplasma* infection *(*ATI*)* in pregnant women. (DOCX)

S2 Table Global, regional and national pooled prevalence of acute *Toxoplasma* infections (ATI) in pregnant women based on strict criteria^⁎^ (results from 82 datasets performed in 47 countries). (DOCX)

S1 Fig Global prevalence of acute *Toxoplasma* infections (ATI) in pregnant women in the Northern and Southern hemispheres. **Abbreviations:** CI, confidence interval; ATI, acute *Toxoplasma* Infections.
(TIF)

S2 Fig Global prevalence of acute *Toxoplasma* infections in pregnant women in the Eastern and Western hemispheres. **Abbreviations:** CI, confidence interval; ATI, acute *Toxoplasma* Infections.
(TIF)

S3 Fig Ecological linear meta-regression analyses of the prevalence of acute *Toxoplasma* infections (ATI) in pregnant women according to: (panel A) sample size showing a non-statistically significant downward trend in prevalence with increasing sample size (*C* = -5. 79e-08; *P*-value = 0.1); (panel B) implementation years of screening showing a non-statistically significant upward trend in prevalence in more recent years (*C* = 0.0007; *P*-value = 0.57); (panel C) country’s income level showing a showing a statistically significant downward trend in prevalence in countries with higher level of income (*C* = -0.005; *P*-value = 0.03); (panel D) human development index (HDI) showing a statistically significant downward trend in prevalence in countries with higher levels of HDI (*C* = -0.005; *P*-value = 0.006); (panel E) geographical latitude a showing a statistically significant downward trend in prevalence with increasing geographical latitude (*C* = -0.00026, *P*-value = 0.005); (panel F) geographical longitude a showing a statistically significant upward trend in prevalence with increasing geographical longitude (*C* = 0.0008, *P*-value = 0.02); (panel G) the mean temperature showing a statistically significant upward trend in prevalence with increasing mean temperature (*C* = 0.0004; *P*-value = 0.02); (panel H) the relative humidity showing a non-statistically significant downward trend in prevalence in areas with higher relative humidity (*C* = -0.0001, *P*-value = 0.18); (panel I) the annual precipitation showing a non-statistically significant downward trend in prevalence with increasing rate of precipitation (*C* = -6.41, *P*-value = 0.81). **Abbreviations:** *C*, coefficient; ES, effect size (Prevalence of ATI).
(TIF)

S1 Text Details of the databases searches. (DOCX)

## References

[1] Montoya JG, Remington JS. Management of *Toxoplasma gondii* infection during pregnancy. Clin Infect Dis. 2008; 47(4):554–66. https://doi.org/1 10.1086/590149 10.1086/590149 . 18624630

[2] Montoya JG, Liesenfeld O. Toxoplasmosis. Lancet. 2004; 363(9425):1965–76. 10.1016/S0140-6736(04)16412-X . 15194258

[3] Torgerson PR, Mastroiacovo P. The global burden of congenital toxoplasmosis: a systematic review. Bull World Health Organ. 2013; 91(7):501–8. 10.2471/BLT.12.111732 . 23825877 PMC3699792

[4] Olariu TR, Remington JS, McLeod R, Alam A, Montoya JG. Severe congenital toxoplasmosis in the United States: clinical and serologic findings in untreated infants. Pediatr Infect Dis J. 2011; 30(12):1056–61. 10.1097/INF.0b013e3182343096 . 21956696

[5] Fallahi S, Rostami A, Shiadeh MN, Behniafar H, Paktinat S. A literature review on maternal-fetal and reproductive disorders of *Toxoplasma gondii* infection. J Gynecol Obstetr Human Reprod. 2017; 47(3):133–140. 10.1016/j.jogoh.2017.12.003 29229361

[6] Brown AS, Schaefer CA, Quesenberry CP Jr, Liu L, Babulas VP, Susser ES. Maternal exposure to toxoplasmosis and risk of schizophrenia in adult offspring. Am J Psychiatr. 2005; 162(4):767–73. 10.1176/appi.ajp.162.4.767 . 15800151

[7] Boyer K, Hill D, Mui E, Wroblewski K, Karrison T, Dubey J, et al Unrecognized ingestion of *Toxoplasma gondii* oocysts leads to congenital toxoplasmosis and causes epidemics in North America. Clin Infect Dis. 2011; 53(11):1081–9. 10.1093/cid/cir667 22021924 PMC3246875

[8] McAuley JB. Congenital toxoplasmosis. J Pediatric Infect Dis Soci. 2014;3(suppl_1):S30–S5. 10.1093/jpids/piu077 . 25232475 PMC4164182

[9] Mandelbrot L, Kieffer F, Sitta R, Laurichesse-Delmas H, Winer N, Mesnard L, et al Prenatal therapy with pyrimethamine+ sulfadiazine vs spiramycin to reduce placental transmission of toxoplasmosis: a multicenter, randomized trial. Am J Obstetr Gynecol. 2018; 219(4):386e1–e9. 10.1016/j.ajog.2018.05.031 . 29870736

[10] Prusa A-R, Kasper DC, Pollak A, Gleiss A, Waldhoer T, Hayde M. The Austrian toxoplasmosis register, 1992–2008. Clin Infect Dis. 2014; 60(2):e4–e10. 10.1093/cid/ciu724 . 25216688

[11] Hotop A, Hlobil H, Groß U. Efficacy of rapid treatment initiation following primary *Toxoplasma gondii* infection during pregnancy. Clin Infect Dis. 2012; 54(11):1545–52. 10.1093/cid/cis234 . 22460980

[12] SYROCOT (Systematic Review on Congenital Toxoplasmosis) study group, Thiébaut R, Leproust S, Chêne G, Gilbert R. Effectiveness of prenatal treatment for congenital toxoplasmosis: a meta-analysis of individual patients' data. Lancet. 2007; 369(9556):115–22. 10.1016/S0140-6736(07)60072-5 . 17223474

[13] Cortina-Borja M, Tan H, Wallon M, Paul M, Prusa A, Buffolano W, et al European Multicentre Study on Congenital Toxoplasmosis (EMSCOT) Prenatal treatment for serious neurological sequelae of congenital toxoplasmosis: an observational prospective cohort study. PLoS Med. 2010; 7(10):1–11. https://doi.org/ 10.1371/journal.pmed.1000351 10.1371/journal.pmed.1000351 20967235 PMC2953528

[14] Wallon M, Peyron F, Cornu C, Vinault S, Abrahamowicz M, Kopp CB, et al Congenital *Toxoplasma* infection: monthly prenatal screening decreases transmission rate and improves clinical outcome at age 3 years. Clin Infect Dis. 2013; 56(9):1223–31. 10.1093/cid/cit032 . 23362291

[15] Dard C, Fricker-Hidalgo H, Brenier-Pinchart M-P, Pelloux H. Relevance of and new developments in serology for toxoplasmosis. Trend Parasitol. 2016; 32(6):492–506. 10.1016/j.pt.2016.04.001 . 27167666

[16] Rostami A, Karanis P, Fallahi S. Advances in serological, imaging techniques and molecular diagnosis of *Toxoplasma gondii* infection. Infection. 2018: 46(3):303–315. 10.1007/s15010-017-1111-3 . 29330674

[17] Maldonado YA, Read JS, Diseases CoI. Diagnosis, treatment, and prevention of congenital toxoplasmosis in the United States. Pediatrics. 2017: 139(2):e20163860 10.1542/peds.2016-3860 . 28138010

[18] Olariu TR, Blackburn BG, Press C, Talucod J, Remington JS, Montoya JG. Role of *Toxoplasma* IgA as part of a Reference Panel for the Diagnosis of Acute Toxoplasmosis During Pregnancy. J Clin Microbiol. 2019: 57(2):e01357–18. 10.1128/JCM.01357-18 30463899 PMC6355551

[19] Montoya JG. Systematic screening and treatment of toxoplasmosis during pregnancy: is the glass half full or half empty? Am J Obstet Gynecol. 2018; 219(4):315–319. 10.1016/j.ajog.2018.08.001 . 30269768

[20] Candolfi E, Pastor R, Huber R, Filisetti D, Villard O. IgG avidity assay firms up the diagnosis of acute toxoplasmosis on the first serum sample in immunocompetent pregnant women. Diag Microbiol Infect Dis. 2007; 58(1):83–8. 10.1016/j.diagmicrobio.2006.12.010 . 17368807

[21] Hedman K, Lappalainen M, Seppäiä I, Mäkelä O. Recent primary *Toxoplasma* infection indicated by a low avidity of specific IgG. J Infect Dis. 1989; 159(4):736–40. 10.1093/infdis/159.4.736 . 2926163

[22] Pomares C, Montoya JG. Laboratory diagnosis of congenital toxoplasmosis. J Clin Microbiol. 2016: 54(10):2448–54. 10.1128/JCM.00487-16 27147724 PMC5035424

[23] Moher D, Shamseer L, Clarke M, Ghersi D, Liberati A, Petticrew M, et al Preferred reporting items for systematic review and meta-analysis protocols (PRISMA-P) 2015 statement. Systematic Rev. 2015; 4(1):1 10.1186/2046-4053-4-1 25554246 PMC4320440

[24] World Health Organization. List of Member States by WHO region and mortality stratum. World Health Report 2003; 2003:182.

[25] World Bank Group database. [Accessed Novamber 2018]. Available from: https://datahelpdesk.worldbank.org/knowledgebase/articles/906519-world-bank-country-and-lending-groups.

[26] United Nations Development Program. [Accessed Novamber 2018]. Available from: http://hdr.undp.org/en/composite/HDI.

[27] DerSimonian R, Laird N. Meta-analysis in clinical trials. Control Clin Trials. 1986; 7(3):177–88. 10.1016/0197-2456(86)90046-2 . 3802833

[28] Higgins JP, Thompson SG, Deeks JJ, Altman DG. Measuring inconsistency in meta-analyses. BMJ. 2003; 327(7414):557–60. 10.1136/bmj.327.7414.557 12958120 PMC192859

[29] Harbord RM, Higgins JP. Meta-regression in Stata. Stata J. 2008; 8(4):493–519. 10.1177/1536867X0800800403.

[30] El Bissati K, Levigne P, Lykins J, Adlaoui EB, Barkat A, Berraho A, et al Global initiative for congenital toxoplasmosis: an observational and international comparative clinical analysis. Emerg Microbes Infect. 2018;7(1):165 10.1038/s41426-018-0164-4 30262847 PMC6160433

[31] Wallon M, Peyron F. Congenital Toxoplasmosis: A Plea for a Neglected Disease. Pathogens. 2018; 7(1):25 10.3390/pathogens7010025 29473896 PMC5874751

[32] Peyron F, Mc Leod R, Ajzenberg D, Contopoulos-Ioannidis D, Kieffer F, Mandelbrot L, et al Congenital toxoplasmosis in France and the United States: One parasite, two diverging approaches. PLoS Negl Trop Dis. 2017; 11(2):e0005222 10.1371/journal.pntd.0005222 28207736 PMC5312802

[33] McLeod R, Boyer K, Karrison T, Kasza K, Swisher C, Roizen N, et al Outcome of treatment for congenital toxoplasmosis, 1981–2004: the national collaborative Chicago-based, congenital toxoplasmosis study. Clin Infect Dis. 2006; 42(10):1383–94. 10.1086/501360 . 16619149

[34] Rostami A, Keshavarz H, Shojaee S, Mohebali M, Meamar AR. Frequency of *Toxoplasma gondii* in HIV positive patients from West of Iran by ELISA and PCR. Iran J Parasitol. 2014; 9(4):474–81. 25759728 PMC4345086

[35] Rostami A, Seyyedtabaei SJ, Aghamolaie S, Behniafar H, Lasjerdi Z, Abdolrasouli A, et al Seroprevalence and risk factors associated with *Toxoplasma gondii* infection among rural communities in northern Iran. Rev Instit Med Trop São Paulo. 2016; 58: 70 10.1590/S1678-9946201658070 27680175 PMC5048641

[36] Wyman CP, Gale SD, Hedges-Muncy A, Erickson LD, Wilson E, Hedges DW. Association between *Toxoplasma gondii* seropositivity and memory function in nondemented older adults. Neurobiol Aging. 2017; 53:76–82. 10.1016/j.neurobiolaging.2017.01.018 28235681 PMC5482532

[37] Dubey J. *Toxoplasma gondii* oocyst survival under defined temperatures. J Parasitol. 1998: 84(4):862–5. 9714227

[38] Frenkel J, Ruiz A, Chinchilla M. Soil survival of *Toxoplasma* oocysts in Kansas and Costa Rica. Am J Trop Med Hyg. 1975; 24(3):439–43. 10.4269/ajtmh.1975.24.439 . 1098494

[39] Nogareda F, Le Strat Y, Villena I, De Valk H, Goulet V. Incidence and prevalence of *Toxoplasma gondii* infection in women in France, 1980–2020: model-based estimation. Epidemiol Infect. 2014; 142(8):1661–70. 10.1017/S0950268813002756 . 24229712 PMC9151203

[40] Gomez CA, Budvytyte LN, Press C, Zhou L, McLeod R, Maldonado Y, et al Evaluation of Three Point-of-Care Tests for Detection of *Toxoplasma* Immunoglobulin IgG and IgM in the United States: Proof of Concept and Challenges. Open Forum Infect Dis. 2018; 5(10):215 10.1093/ofid/ofy215 30393749 PMC6204989

[41] Lykins J, Li X, Levigne P, Zhou Y, El Bissati K, Clouser F, et al Rapid, inexpensive, fingerstick, whole-blood, sensitive, specific, point-of-care test for anti-*Toxoplasma* antibodies. PLoS Negl Trop Dis. 2018; 12(8):e0006536 10.1371/journal.pntd.0006536 30114251 PMC6095485

[42] Begeman IJ, Lykins J, Zhou Y, Lai BS, Levigne P, El Bissati K, et al Point-of-care testing for *Toxoplasma gondii* IgG/IgM using *Toxoplasma* ICT IgG-IgM test with sera from the United States and implications for developing countries. PLoS Negl Trop Dis. 2017; 11(6):e0005670 10.1371/journal.pntd.0005670 28650970 PMC5501679

