# Supplementary material for: Acute Toxoplasma infection in pregnant women worldwide: A systematic review and meta-analysis
Source: PLoS Negl Trop Dis. 2019 Oct 14;13(10):e0007807. doi: 10.1371/journal.pntd.0007807 (PMC6822777; doi:10.1371/journal.pntd.0007807)
Supplement: S1 Table — (DOCX) [file pntd.0007807.s002.docx]

**­­­S1 Table.** Main characteristics of all eligible studies reporting prevalence of acute *Toxoplasma* infection *(*ATI*)* in pregnant women

| WHO region/primary author [reference] | Publication year | Study period | Country | Human development index (HDI) | Income level of country | Mean temperature (°C) | Rain fall | Humidity (%) | Longitude | Latitude | Methods for diagnosis of ATI | Number of pregnant women screened (total) | Number of pregnant women with of ATI |
| --- | --- | --- | --- | --- | --- | --- | --- | --- | --- | --- | --- | --- | --- |
| Eastern Mediterranean region |  |  |  |  |  |  |  |  |  |  |  |  |  |
| Eshratkhah Mohammadnejad [1] | 2018 | 2015-2016 | Iran | High | Upper Middle | 11 | 390 | 58 | 45.07 E | 37.54 N | ELISA # | 620 | 3 |
| Fallahizadeh [2] | 2018 | 2016 | Iran | High | Upper Middle | 24 | 312 | 36 | 48.1 E | 32.1 N | ELISA # | 276 | 2 |
| Rehman [3] | 2018 | 2012-2013 | Pakistan | Medium | Lower Middle | 22 | 560 | 60 | 72 E | 34.1 N | ELISA # | 180 | 0 |
| Sardarian [4] | 2018 | 2016-2017 | Iran | High | Upper Middle | 12 | 400 | 43 | 48.3 E | 34.4 N | ELISA # | 653 | 2 |
| Naghili [5] | 2017 | 2010-2013 | Iran | High | Upper Middle | 12 | 318 | 55 | 46.27 E | 38.1 N | ELISA # | 391 | 58 |
| Alanazi [6] | 2017 | 2015-2016 | Saudi Arabia | Very high | High | 22 | 96 | 36 | 41.05 E | 30.95 N | ELISA # | 340 | 2 |
| Laboudi [7] | 2017 | 2015-2016 | Morocco | Medium | Lower Middle | 18 | 523 | 80 | 6.51 W | 34.02 N | ELISA # | 128 | 5 |
| Mahdy [8] | 2017 | 2012-2014 | Yemen | Low | Lower Middle | 16 | 378 | 70 | 44.01 E | 13.57 N | ELISA # | 359 | 5 |
| Nahouli* [9] | 2017 | 1994-2015 | Lebanon | High | Upper Middle | 25 | 585 | 69 | 35.50 E | 33.89 N | ELISA ⁂ | 2456 | 46 |
| Norouzi Larki [10] | 2017 | 2014 | Iran | High | Upper Middle | 17 | 316 | 41 | 52.58 E | 29.59 N | ELISA # | 2000 | 5 |
| Shieh [11] | 2017 | 2015 | Iran | High | Upper Middle | 12 | 316 | 46 | 49.70 E | 34.09 N | ELISA # | 261 | 2 |
| Ahmadpour [12] | 2017 | 2012-2013 | Iran | High | Upper Middle | 20 | 369 | 62 | 47.58 E | 34.04 N | ELISA # | 264 | 15 |
| Garedaghi [13] | 2017 | 2014 | Iran | High | Upper Middle | 12 | 405 | 51 | 46.1 E | 36.9 N | ELISA # | 200 | 5 |
| Shirdel [14] | 2017 | 2014-2015 | Iran | High | Upper Middle | 13 | 511 | 63 | 55.13 E | 37.28 N | ELISA # | 440 | 9 |
| Akhlaghi [15] | 2016 | 2014 | Iran | High | Upper Middle | 14 | 256 | 48 | 50.93 E | 35.84 N | ELISA # | 468 | 9 |
| Al-Eryani [16] | 2016 | 2010-2011 | Yemen | Low | Lower Middle | 16 | 265 | 36 | 48.51 E | 15.55 N | ELISA # | 593 | 44 |
| Alghamdi [17] | 2016 | 2011 | Saudi Arabia | Very high | High | 25 | 11 | 26 | 46.67 E | 24.71 N | ELISA # | 250 | 16 |
| Mohaghegh [18] | 2016 | 2013-2015 | Iran | High | Upper Middle | 13 | 279 | 58 | 57.31 E | 37.47 N | ELISA # | 350 | 12 |
| Mohamed [19] | 2016 | 2014 | Saudi Arabia | Very high | High | 30 | 70 | 46 | 39.85 E | 21.38 N | ELISA # | 326 | 5 |
| Rostamzadeh Khameneh [20] | 2016 | 2014 | Iran | High | Upper Middle | 11 | 390 | 61 | 45.07 E | 37.54 N | ELISA # | 156 | 4 |
| Bassiouny [21] | 2016 | 2015-2016 | Egypt | Medium | Lower Middle | 21 | 183 | 67 | 29.55 E | 31.12 N | ELISA # | 382 | 2 |
| Imam [22] | 2016 | 2014-2015 | Saudi Arabia | Very high | High | 28 | 41 | 25 | 39.56 E | 24.52 N | ELISA # | 150 | 0 |
| Tabatabaei [23] | 2015 | 2013-2014 | Iran | High | Upper Middle | 14 | 301 | 49 | 50.54 E | 36.04 N | ELISA # | 200 | 4 |
| Elsafi [24] | 2015 | 2012-2013 | Saudi Arabia | Very high | High | 26 | 80 | 55 | 50.03 E | 26.23 N | MEIA # | 400 | 12 |
| Kamal [25] | 2015 | 2013-2014 | Egypt | Medium | Lower Middle | 20 | 31 | 52 | 30.76 E | 28.08 N | ELISA # | 120 | 0 |
| Abdel-Raouff [26] | 2014 | 2013 | Sudan | Low | Lower Middle | 29 | 135 | 25 | 32.55 E | 15.5 N | ELISA # | 163 | 0 |
| Sharbatkhori [27] | 2014 | 2012 | Iran | High | Upper Middle | 13 | 511 | 70 | 54.43 E | 36.84 N | ELISA # | 555 | 8 |
| Yad Yad [28] | 2014 | 2011 | Iran | High | Upper Middle | 24 | 250 | 45 | 48.29 E | 30.34 N | ELISA # | 501 | 3 |
| Aqeely [29] | 2014 | 2013 | Saudi Arabia | Very high | High | 30 | 106 | 68 | 42.57 E | 16.88 N | ELISA # | 195 | 4 |
| Ghasemloo [30] | 2014 | 2010-2013 | Iran | High | Upper Middle | 17 | 220 | 46 | 51.38 E | 35.68 N | ELISA # | 785 | 0 |
| Ahmed [31] | 2014 | 2013-2014 | Egypt | Medium | Lower Middle | 20 | 33 | 55 | 31.71 E | 30.73 N | ELISA # | 100 | 8 |
| Ashankyty [32] | 2014 | 2013 | Saudi Arabia | Very high | High | 21 | 174 | 25 | 39.12 E | 25.11 N | ELISA # | 6076 | 35 |
| Almushait [33] | 2014 | 2008-2010 | Saudi Arabia | Very high | High | 18 | 278 | 54 | 42.51 E | 18.24 N | ELISA # | 487 | 16 |
| Ben Abdallah [34] | 2013 | 2007-2011 | Tunisia | High | Lower Middle | 18 | 448 | 77 | 9.5 E | 33.8 N | ELISA and IgG Avidity ⁋ | 2070 | 53 |
| Akhlaghi [35] | 2013 | 2010 | Iran | High | Upper Middle | 14 | 256 | 41 | 50.87 E | 34.63 N | ELISA # | 200 | 11 |
| Babaie [36] | 2013 | 2010-2011 | Iran | High | Upper Middle | 14 | 251 | 54 | 59.61 E | 36.26 N | ELISA # | 419 | 27 |
| El Deeb [37] | 2012 | 2010-2011 | Egypt | Medium | Lower Middle | 21 | 34 | 62 | 30.93 E | 30.46 N | ELISA and IgG Avidity ⁋ | 3 23 | 1 |
| Elamin [38] | 2012 | 2010-2011 | Sudan | Low | Lower Middle | 28 | 342 | 35 | 33.53 E | 14.39 N | ELISA # | 94 | 6 |
| Hajsoleimani [39] | 2012 | 2010-2011 | Iran | High | Upper Middle | 11 | 419 | 53 | 48.5 E | 36.68 N | ELISA # | 500 | 7 |
| Bin Dajem [40] | 2012 | 2009-2010 | Saudi Arabia | Very high | High | 18 | 278 | 69 | 42.86 E | 19.09 N | ELISA # | 137 | 9 |
| Almogren [41] | 2011 | 2009-2010 | Saudi Arabia | Very high | High | 25 | 111 | 26 | 46.67 E | 24.71 N | ELISA # | 2176 | 0 |
| Mohammad [42] | 2010 | 2009 | Saudi Arabia | Very high | High | 26 | 74 | 39 | 50.67 E | 22.29 N | ELISA # | 554 | 11 |
| Al-Nahari [43] | 2010 | 2007-2008 | Yemen | Low | Lower Middle | 16 | 265 | 36.5 | 44.19 E | 15.36 N | ELISA # | 463 | 55 |
| Parvizpour [44] | 2010 | 2008 | Iran | High | Upper Middle | 13 | 502 | 76 | 46.93 E | 34.79 N | ELISA # | 201 | 33 |
| Iqbal [45] | 2007 | 2002-2005 | Kuwaiti | Very high | High | 26 | 103 | 60 | 47.48 E | 29.31 N | ELISA and IgG Avidity ⁋ | 224 | 9 |
| Saeedi [46] | 2007 | 2004 | Iran | High | Upper Middle | 14 | 511 | 76 | 54.43 E | 36.84 N | ELISA # | 300 | 29 |
| Al-Harthi [47] | 2006 | 2004 | Saudi Arabia | Very high | High | 30 | 70 | 46 | 39.85 E | 21.38 N | ELISA # | 197 | 11 |
| Elnahas [48] | 2003 | 2000 | Sudan | Low | Lower Middle | 30 | 135 | 29 | 32.55 E | 15.5 N | ELISA # | 487 | 5 |
| Noorbakhsh [49] | 2002 | 1999-2000 | Iran | High | Upper Middle | 16 | 220 | 43 | 51.38 E | 35.68 N | ELISA # | 140 | 9 |
| Al-Mulhim [50] | 2001 | 1999-2000 | Saudi Arabia | Very high | High | 26 | 77 | 41 | 49.97 E | 26.39 N | MEIA # | 175 | 1 |
| Dar [51] | 1997 | 1995-1996 | United Arab Emirates | Very high | high | 29 | 72 | 63 | 54.37 E | 24.45 N | ELISA # | 301 | 47 |
| El-Nawawy* [52] | 1996 | 1992-1993 | Egypt | Medium | Upper Middle | 21 | 30 | 55 | 31.2 E | 3.4 N | ELISA # | 150 | 3 |
| Franklin* [53] | 1993 | 1991-1992 | Israel | Very high | High | 21 | 558 | 59 | 35.5 E | 32.7 N | ELISA, IFA # | 213 | 3 |
| African Region |  |  |  |  |  |  |  |  |  |  |  |  |  |
| Paul [54] | 2018 | 2017 | Tanzania | Low | Low | 26.6 | 1138 | 71 | 37.4 E | 3.5 S | ELISA # | 254 | 23 |
| Berredjem [55] | 2017 | 2015-2016 | Algeria | High | Upper Middle | 18 | 706 | 60 | 7.7 E | 36.8 N | ELISA and IgG Avidity ⁋ | 143 | 9 |
| Murebwayire [56] | 2017 | 2014 | Rwanda | Low | Low | 20 | 1000 | 73 | 30.10 E | 1.97 S | ELISA # | 384 | 15 |
| Yohanes [57] | 2017 | 2015 | Ethiopia | Low | Low | 22 | 818 | 45 | 37.56 E | 6.02 N | ELISA # | 232 | 2 |
| Bamba [58] | 2017 | 2013-2014 | Burkina Faso | Low | Low | 27 | 1051 | 55 | 4.3 W | 11.16 N | ELISA and IgG Avidity ⁋ | 316 | 0 |
| Frimpong [59] | 2017 | 2015 | Zambia | Low | Lower Middle | 20 | 831 | 67 | 28.32 E | 15.38 S | LFIA # | 411 | 0 |
| Volker [60] | 2017 | 2011-2012 | Ghana | Medium | Lower Middle | 26 | 809 | 83 | 1.02 W | 7.94 N | ELISA and IgG Avidity ⁋ | 168 | 0 |
| Lobo [61] | 2017 | 2011 | Angola | Medium | Upper Middle | 24 | 439 | 79 | 13.14 E | 8.50 S | ELISA and IgG Avidity ⁋ | 300 | 0 |
| Abamecha [62] | 2016 | 2014-2015 | Ethiopia | Low | Low | 16 | 1675 | 56 | 35.56 E | 6.63 N | ELISA # | 232 | 7 |
| Ayi [63] | 2016 | 2014-2015 | Ghana | Medium | Lower Middle | 26 | 809 | 81 | 0.18 W | 5.6 N | ELISA # | 125 | 0 |
| Nasir [64] | 2015 | 2011-2013 | Nigeria | Low | Lower Middle | 26 | 613 | 23 | 13.15 E | 11.83 N | ELISA and IgG Avidity ⁋ | 360 | 26 |
| Messerer [65] | 2014 | 2006-2009 | Algeria | High | Upper Middle | 18 | 712 | 85 | 7.75 E | 36.92 N | ELISA # | 1028 | 11 |
| [Doudou](https://www.sciencedirect.com/science/article/pii/S2221169114602112#!) [66] | 2014 | 2011 | Congo | Medium | Low | 25 | 1368 | 80 | 15.26 E | 4.44 S | ELISA and IgG Avidity ⁋ | 378 | 2 |
| De Paschale [67] | 2014 | 2011 | Benin | Low | Low | 27 | 1200 | 80 | 1.67 E | 10.79 N | ELISA and IgG Avidity ⁋ | 283 | 0 |
| Mwambe [68] | 2013 | 2012-2013 | Tanzania | Low | Low | 23 | 1054 | 64 | 32.71 E | 2.87 S | ELISA # | 350 | 0 |
| Zemene [69] | 2012 | 2011 | Ethiopia | Low | Low | 19 | 1624 | 72 | 36.83 E | 7.67 N | ELISA # | 201 | 5 |
| Linguissi [70] | 2012 | 2006-2009 | Burkina Faso | Low | Low | 28 | 1000 | 48 | 1.51 E | 12.37 N | ELISA # | 182 | 7 |
| Njunda [71] | 2011 | 2009 | Cameroom | Medium | Lower Middle | 26 | 3702 | 90 | 9.76 E | 4.05 N | EIA # | 110 | 3 |
| Ndiaye* [72] | 2011 | 2002-2006 | Senegal | Low | Low | 25 | 469 | 78 | 17.4 W | 14.7 N | ELISA # | 941 | 96 |
| Sitoe [73] | 2010 | 2008-2009 | Mozambique | Low | Low | 23 | 781 | 65 | 32.6 E | 25.89 S | ELISA # | 150 | 1 |
| Mpiga Mickoto [74] | 2010 | 2007 | Gabon | High | Upper Middle | 25 | 1827 | 84 | 13.6 E | 1.61 S | ELFA # | 839 | 22 |
| Ayi [75] | 2009 | 2007-2008 | Ghana | Medium | Lower Middle | 27 | 809 | 81 | 0.18 W | 5.6 N | ELISA # | 159 | 13 |
| Ishaku [76] | 2009 | 2007-2008 | Nigeria | Low | Lower Middle | 28 | 1050 | 69 | 7.719 E | 11.08 N | EIA # | 374 | 3 |
| Ndiaye* [77] | 2007 | 2002 | Senegal | Low | Low | 25 | 469 | 78 | 17.4 W | 14.7 N | ELISA ⁂ | 109 | 12 |
| Simpore [78] | 2006 | 2004-2005 | Burkina Faso | Low | Low | 28 | 788 | 52 | 1.51 W | 12.37 N | ELISA # | 336 | 0 |
| Diallo [79] | 1996 | 1994-1995 | Senegal | Low | Low | 25 | 469 | 78 | 17.4 W | 14.7 N | ELISA # | 414 | 0 |
| Rodier [80] | 1995 | 1993-1994 | Benin | Low | Low | 26 | 1244 | 83 | 2.3 E | 6.3 N | ELISA # | 211 | 0 |
| Western Pacific region |  |  |  |  |  |  |  |  |  |  |  |  |  |
| Jiang [81] | 2018 | 2016-2017 | China | High | Upper Middle | 5 | 679 | 63 | 126.1 E | 43.4 N | ELISA # | 311 | 4 |
| Zhou [82] | 2015 | 2011-2014 | China | High | Upper Middle | 16 | 1008 | 78 | 120.31 E | 31.49 N | ELISA # | 3014 | 49 |
| Cong [83] | 2015 | 2011-2013 | China | High | Upper Middle | 13 | 730 | 65 | 122.1 E | 37.51 N | ELISA # | 965 | 25 |
| Hung [84] | 2015 | 2009-2011 | Taiwan | High | High | 25 | 1285 | 76 | 121.5 E | 25.03 N | ELISA # | 104 | 1 |
| Chen [85] | 2014 | 2010-2013 | China | High | Upper Middle | 17 | 900 | 75 | 120.31 E | 31.49 N | ELISA # | 2993 | 43 |
| Andiappan [86] | 2014 | 2012-2013 | Malaysia | Very high | Upper Middle | 27 | 2486 | 81 | 101.68 E | 3.13 N | ELISA and IgG Avidity ⁋ | 219 | 0 |
| Zhang [87] | 2014 | 2010-2013 | China | High | Upper Middle | 12 | 612 | 63 | 115.97 E | 39.48 N | ELISA # | 2740 | 44 |
| Emelia [88] | 2014 | 2012-2013 | Malaysia | Very high | Upper Middle | 26 | 2710 | 86 | 101.4 E | 3.8 N | ELISA and IgG Avidity ⁋ | 281 | 1 |
| Hua [89] | 2013 | 2011-2012 | China | High | Upper Middle | 16 | 1050 | 68 | 119.78 E | 33.14 N | ELISA # | 1081 | 0 |
| Sakikawa* [90] | 2011 | 2002-2010 | Japan | Very high | High | 18 | 2547 | 65 | 130 E | 31 N | ELISA ⁂ | 2969 | 3 |
| Chou [91] | 2011 | 2009-2010 | Taiwan | High | high | 21 | 1801 | 77 | 120.6 E | 24.14 N | ELISA # | 220 | 6 |
| Xia [92] | 2011 | 2009-2010 | China | High | Upper Middle | 16 | 1001 | 52 | 118.79 E | 32.1 N | ELISA # | 6849 | 87 |
| Huang [93] | 2010 | 2008-2010 | China | High | Upper Middle | 16 | 1100 | 65 | 104.13 E | 29.9 N | ELISA # | 720 | 12 |
| Liu [94] | 2009 | 2006 | China | High | Upper Middle | 5 | 626 | 63 | 125.32 E | 43.81 N | ELISA # | 235 | 0 |
| Lin [95] | 2008 | 2006-2007 | Taiwan | High | High | 21 | 1801 | 77 | 120.4 E | 24.09 N | ELISA # | 426 | 11 |
| Hu [96] | 2006 | 2004-2005 | Taiwan | High | Upper Middle | 21 | 1801 | 79 | 120.96 E | 23.69 N | ELISA # | 483 | 5 |
| Song [97] | 2005 | 2000-2003 | South Korea | Very high | High | 11 | 1373 | 71 | 126.5 E | 37.5 S | ELISA, PCR # | 5725 | 1 |
| Morris [98] | 2004 | 2002-2003 | New Zealand | Very high | High | 15 | 1284 | 80 | 174.76 E | 36.84 S | EIA # | 500 | 12 |
| Buchy [99] | 2003 | 2001-2002 | Vietnam | Medium | Lower Middle | 27 | 1868 | 75 | 106.6 E | 10.8 N | ELISA # | 300 | 0 |
| Zhang [100] | 1996 | 1994-1995 | China | High | Upper Middle | 21 | 855 | 66 | 113.38 E | 34.29 N | Capture-EIA # | 4126 | 139 |
| Sun [101] | 1995 | 1993-1994 | China | High | Upper Middle | 17 | 961 | 65 | 104.1 E | 30.57 N | ELISA # | 1211 | 48 |
| Walpole [102] | 1991 | 1986-1989 | Australia | Very high | High | 18 | 807 | 63 | 115.8 E | 31.9 S | ELISA # | 10207 | 11 |
| Sfameni [103] | 1988 | 1983 | Australia | Very high | High | 14.8 | 666 | 69 | 144 E | 37.8 S | ELISA # | 609 | 3 |
| Americas Region |  |  |  |  |  |  |  |  |  |  |  |  |  |
| Barbosa Costa [104] | 2018 | 2009-2010 | Brazil | High | Upper Middle | 24 | 1946 | 79 | 39 W | 14.4 S | ELISA # | 726 | 1 |
| Avelar [105] | 2017 | 2014 | Brazil | High | Upper Middle | 25 | 1781 | 65 | 38.5 W | 12.97 S | ELISA # | 712 | 0 |
| Alvarado-Esquivel [106] | 2016 | 2014-2016 | Mexico | High | Upper Middle | 18 | 533 | 58 | 102.3 W | 21.88 N | ELISA # | 338 | 1 |
| Dubey [107] | 2016 | 2009-2011 | Caribbean islands | High | Upper Middle | 17 | 1200 | 83 | 78.65 W | 21.46 N | ELISA # | 437 | 0 |
| da Silva* [108] | 2015 | 2012-2014 | Brazil | High | Upper Middle | 23 | 1303 | 71 | 49.06 W | 11.72 S | ELISA and IgG Avidity ⁋ | 487 | 26 |
| de Quadros [109] | 2015 | 2008-2009 | Brazil | High | Upper Middle | 14 | 1441 | 80 | 50°19 W | 27°48 S | ELISA # | 148 | 1 |
| da Rocha [110] | 2015 | 2013-2014 | Brazil | High | Upper Middle | 23 | 1303 | 60 | 48.29 W | 10.17 S | ELISA # | 338 | 0 |
| Ferezin [111] | 2013 | 2010 | Brazil | High | Upper Middle | 23 | 927 | 80 | 49.26 W | 25.42 S | MEIA # | 1,534 | 17 |
| Fonseca [112] | 2012 | 2007-2008 | Brazil | High | Upper Middle | 21 | 1313 | 72 | 44.89 W | 20.14 S | ELISA # | 2,136 | 77 |
| Bittencourt [113] | 2012 | 2010-2011 | Brazil | High | Upper Middle | 19 | 1325 | 65 | 52.02 W | 25.25 S | ELISA ⁂ | 4022 | 0 |
| Dias [114] | 2011 | 2007-2008 | Brazil | High | Upper Middle | 20 | 1476 | 65 | 51.37 W | 23.31 S | MEIA # | 607 | 6 |
| Sartor [115] | 2011 | 2008 | Brazil | High | Upper Middle | 23 | 1414 | 63 | 49.2 W | 16.6 S | ELISA # | 10316 | 75 |
| Sroka [116] | 2010 | 2005 | Brazil | High | Upper Middle | 26 | 1448 | 78 | 38.52 W | 3.73 S | MEIA # | 963 | 5 |
| Vaz [117] | 2010 | 2003-2004 | Brazil | High | Upper Middle | 21 | 1600 | 80 | 49.26 W | 25.42 S | ELISA and IgG Avidity ⁋ | 20389 | 47 |
| Alarcon de Noya [118] | 2010 | 2008-2009 | Venezuela | High | High | 21 | 933 | 82 | 66.9 W | 10.48 N | ELISA and IgG Avidity ⁋ | 678 | 10 |
| Gonçalves [119] | 2010 | 2006-2007 | Brazil | High | Upper Middle | 19 | 1340 | 78 | 46.63 W | 23.55 S | ELISA # | 556 | 19 |
| Varella [120] | 2009 | 1998-2005 | Brazil | High | Upper Middle | 19 | 1397 | 78 | 51.1 W | 30 S | ELISA and IgG Avidity ⁋ | 41112 | 199 |
| Lopes [121] | 2009 | 2006 | Brazil | High | Upper Middle | 21 | 1429 | 71 | 51.16 W | 23.30 S | ELISA and IgG Avidity ⁋ | 492 | 0 |
| Mauro-Madi [122] | 2009 | 2007-2008 | Brazil | High | Upper Middle | 17 | 1908 | 71 | 51.17 W | 29.16 S | Capture EIA # | 1510 | 27 |
| Alvarado-Esquivel [123] | 2009 | 2007-2008 | Mexico | High | Upper Middle | 18 | 463 | 64 | 104.4 W | 24.01 N | ELISA # | 439 | 0 |
| Barbosa [124] | 2009 | 2007 | Brazil | High | Upper Middle | 26 | 1464 | 79 | 35.2 W | 5.77 S | ELISA and IgG Avidity ⁋ | 190 | 0 |
| Carral [125] | 2008 | 2006-2007 | Argentina | Very high | High | 17 | 1040 | 66 | 58.3 W | 34.6 S | ELISA ⁂ | 13632 | 121 |
| Ramsewak* [126] | 2008 | 2002-2003 | Trinidad and Tobago | High | High | 28 | 1700 | 82 | 61.22 W | 10.69 N | ELISA # | 450 | 34 |
| Rosso [127] | 2008 | 2005 | Colombia | High | Upper Middle | 24 | 1173 | 74 | 76.53 W | 3.45 N | MEIA # | 955 | 27 |
| Ribeiro [128] | 2008 | 2003-2006 | Brazil | High | Upper Middle | 23 | 1274 | 79 | 42.13 W | 21.39 S | ELISA and IgG Avidity ⁋ | 832 | 1 |
| Porto [129] | 2008 | 2004-2005 | Brazil | High | Upper Middle | 26 | 1804 | 80 | 34.87 W | 8.04 S | IFA # | 503 | 14 |
| Castilho-Pelloso [130] | 2007 | 2001-2003 | Brazil | High | Upper Middle | 18 | 2034 | 80 | 49.26 W | 25.42 S | ELISA # | 16,686 | 264 |
| Alvarado-Esquivel [131] | 2006 | 2005-2006 | Mexico | High | Upper Middle | 18 | 463 | 64 | 104.4 W | 24.01 N | MEIA # | 343 | 0 |
| Spalding [132] | 2005 | 1997-1998 | Brazil | High | Upper Middle | 18 | 1205 | 75 | 51.21 W | 30.03 S | ELISA # | 2126 | 77 |
| Tolêdo Nóbrega [133] | 2005 | 2002-2004 | Brazil | High | Upper Middle | 21 | 1668 | 67 | 47.9 W | 15.8 S | ELISA # | 2636 | 15 |
| Avelino [134] | 2003 | 1997-1999 | Brazil | High | Upper Middle | 23 | 1414 | 74 | 49.2 W | 16.6 S | ELISA # | 522 | 45 |
| Gomez-Marin [135] | 1997 | 1994-1996 | Colombia | High | Upper Middle | 19 | 2330 | 75 | 75.6 W | 4.46 N | ISAGA # | 937 | 15 |
| González-Morales* [136] | 1995 | 1990-1991 | Cuba | High | Upper Middle | 25 | 1244 | 74 | 82.3 W | 23.1 N | ELISA ⁂ | 1606 | 16 |
| South East Asia Region |  |  |  |  |  |  |  |  |  |  |  |  |  |
| Naheen [137] | 2018 | 2015-2016 | Bangladesh | Medium | Lower Middle | 26 | 2022 | 74 | 90.2 E | 23.4 N | ELISA # | 150 | 6 |
| Stephen [138] | 2017 | 2015-2017 | India | Medium | Lower Middle | 28 | 1171 | 80 | 79.8 E | 11.9 N | ELISA and IgG Avidity ⁋ | 193 | 2 |
| van Enter [139] | 2017 | 2014-2015 | Thailand | High | Upper Middle | 27 | 1737 | 78 | 100.50 E | 13.75 N | ELISA and IgG Avidity ⁋ | 199 | 3 |
| Iddawela [140] | 2017 | 2010-2013 | Sri Lanka | High | Lower Middle | 25 | 2132 | 84 | 80.59 E | 7.269 N | ELISA # | 536 | 2 |
| Chandrasena [141] | 2016 | 2014 | Sri Lanka | High | Lower Middle | 27 | 2398 | 74 | 79.87 E | 7.2 N | LFIA # | 293 | 0 |
| Amar [142] | 2015 | 2012-2013 | India | Medium | Lower Middle | 26 | 981 | 59 | 81.84 E | 25.43 N | ELISA # | 103 | 2 |
| Singh [143] | 2014 | 2011-2012 | India | Medium | Lower Middle | 25 | 1000 | 63 | 78.5 E | 20.5 N | ELISA and IgG Avidity ⁋ | 751 | 3 |
| Andiappan [86] | 2014 | 2012-2013 | Myanmar | Medium | Lower Middle | 27 | 2738 | 74 | 96.19 E | 16.86 N | ELISA and IgG Avidity ⁋ | 215 | 0 |
| Andiappan [144] | 2014 | 2012-2013 | Thailand | High | Upper Middle | 27 | 1877 | 81 | 100.4 E | 7 N | ELISA # | 760 | 0 |
| Malarvizhi [145] | 2012 | 2009-2010 | India | Medium | Lower Middle | 29 | 1197 | 73 | 80.2 E | 13 N | ELISA # | 232 | 9 |
| Nissapatorn [146] | 2011 | 2009-2010 | Thailand | High | Upper Middle | 27 | 1877 | 80 | 100.4 E | 7.00 N | ELISA # | 640 | 0 |
| Khurana* [147] | 2010 | 2005-2006 | India | Medium | Lower Middle | 24 | 979 | 54 | 77.12 E | 28.12 N | ELISA # | 300 | 9 |
| Singh* [148] | 2004 | 2002-2003 | India | Medium | Lower Middle | 25 | 693 | 54 | 77.20 E | 28.61 N | ELISA # | 300 | 2 |
| Wanachiwanawin [149] | 2001 | 1998-2000 | Thailand | High | Upper Middle | 29 | 1611 | 78 | 100.9 E | 15.8 N | ELISA # | 831 | 2 |
| Tantivanich [150] | 2001 | 1999-2000 | Thailand | High | Upper Middle | 29 | 1611 | 79 | 100.9 E | 15.87 N | ELISA # | 200 | 11 |
| Sukthana [151] | 1999 | 1997-1998 | Thailand | High | Upper Middle | 28 | 1498 | 78 | 100.2 E | 13.54 N | ISAGA # | 300 | 0 |
| Ashrafunnessa [152] | 1998 | 1995-1996 | Bangladesh | Medium | Lower Middle | 26 | 1875 | 81 | 90.41 E | 23.81 N | ELISA # | 88 | 1 |
| Taechowisan [153] | 1997 | 1992-1995 | Thailand | High | Upper Middle | 29 | 1611 | 78 | 100.4 E | 13.76 N | ELISA # | 300 | 3 |
| European Region |  |  |  |  |  |  |  |  |  |  |  |  |  |
| Tanrıverdi [154] | 2018 | 2013-2016 | Turkey | High | Upper Middle | 7 | 476 | 67 | 41.1 E | 39.5 N | ELISA and IgG Avidity ⁋ | 25525 | 10 |
| Sirin [155] | 2017 | 2014-2016 | Turkey | High | Upper Middle | 17 | 687 | 58 | 27.14 E | 38.42 N | ELISA and IgG Avidity ⁋ | 7513 | 6 |
| Lobo [61] | 2017 | 2010-2011 | Portugal | Very high | High | 17 | 691 | 74 | 9.8 W | 38.43 N | ELISA and IgG Avidity ⁋ | 155 | 0 |
| Cetin [156] | 2017 | 2009-2016 | Turkey | High | Upper Middle | 18 | 591 | 63 | 36.1 E | 36.2 N | ELISA ⁂ | 11564 | 93 |
| Liassides [157] | 2016 | 2009-2011 | Cyprus | Very high | High | 18 | 329 | 56 | 33.3 E | 35.1 N | ELISA # | 17631 | 107 |
| Berghold [158] | 2016 | 1995-2001 | Austria | Very high | High | 9 | 849 | 92 | 14.4 E | 47.3 N | ELISA, VIDAS ⁂ | 103316 | 880 |
| Lange [159] | 2016 | 2002-2008 | Germany | Very high | High | 8 | 560 | 75 | 13.39E | 54.08 N | ELISA and IgG Avidity ⁋ | 5402 | 17 |
| Billi [160] | 2016 | 2012-2014 | Italy | Very high | high | 12 | 903 | 77 | 11.21 E | 44.59 N | ELISA and IgG Avidity ⁋ | 36877 | 71 |
| Burak Selek [161] | 2015 | 2012-2014 | Turkey | High | Upper Middle | 14 | 747 | 72 | 28.9 E | 41 N | ELISA and IgG Avidity ⁋ | 1737 | 1 |
| Parlak [162] | 2015 | 2012-2013 | Turkey | High | Upper Middle | 9 | 409 | 61 | 43.37 E | 38.50N | ELISA and IgG Avidity ⁋ | 9809 | 19 |
| Aynioglu [163] | 2015 | 2012-2014 | Turkey | High | Upper Middle | 14 | 1113 | 74 | 31.78 E | 41.45 N | ELISA # | 910 | 23 |
| Gundem [164] | 2014 | 2013 | Turkey | High | Upper Middle | 13 | 794 | 58 | 32.49 E | 37.87 N | ELISA # | 410 | 1 |
| Karacan [165] | 2014 | 2009-2013 | Turkey | High | Upper Middle | 14 | 747 | 68 | 28.97 E | 41.00 N | ELISA and IgG Avidity ⁋ | 1258 | 1 |
| Gencer [166] | 2014 | 2012-2013 | Turkey | High | Upper Middle | 15 | 637 | 68 | 26.41 E | 40.11 N | ELISA # | 196 | 5 |
| Mumcuoglu [167] | 2014 | 2010-2013 | Turkey | High | Upper Middle | 15 | 750 | 68 | 32.85 E | 39.93 N | ELISA and IgG Avidity ⁋ | 4758 | 4 |
| Nowakowska [168] | 2014 | 2004-2012 | Poland | Very high | High | 7 | 676 | 45 | 19.45 E | 51.75 N | ELISA and IgG Avidity ⁋ | 8281 | 49 |
| Uysal [169] | 2013 | 2011-2012 | Turkey | High | Upper Middle | 17 | 687 | 68 | 27.14 E | 38.42 N | ELISA and IgG Avidity ⁋ | 4651 | 1 |
| Jerant-Patic [170] | 2013 | 2010-2012 | Serbia | High | Upper Middle | 12 | 647 | 66 | 19.8 E | 45.2 N | ELISA # | 662 | 5 |
| Dogan [171] | 2012 | 2010-2011 | Turkey | High | Upper Middle | 19 | 409 | 68 | 38.33 E | 38.35 N | ELISA # | 312 | 0 |
| Dentico [172] | 2011 | 2005 | Kosovo | High | Lower Middle | 12 | 983 | 71 | 21.1 E | 42.4 N | ELISA and IgG Avidity ⁋ | 334 | 4 |
| Karabulut [173] | 2011 | 2008-2009 | Turkey | High | Upper Middle | 13 | 794 | 68 | 29.23 E | 37.61 N | ELISA and IgG Avidity ⁋ | 1102 | 9 |
| Sagel [174] | 2011 | 2000-2007 | Austria | Very high | High | 9 | 852 | 85 | 14.1 E | 48.1 N | ELISA ⁂ | 63416 | 66 |
| de Paschale [175] | 2010 | 2006-2008 | Italy | Very high | High | 12 | 1129 | 75 | 8.90 E | 45.59 N | ELISA and IgG Avidity ⁋ | 4694 | 14 |
| Maggi [176] | 2009 | 2004-2005 | Albania | High | Upper Middle | 17 | 1250 | 71 | 19.81 E | 41.32 N | ELISA and IgG Avidity ⁋ | 496 | 2 |
| Tamer [177] | 2009 | 2005-2007 | Turkey | High | Upper Middle | 14 | 692 | 75 | 28.97 E | 41 N | ELISA # | 1972 | 31 |
| Dolgikh [178] | 2008 | 1992-2006 | Russia | Very high | High | 1.3 | 393 | 71 | 73.3 E | 54.9 N | ELISA and IgG Avidity ⁋ | 9365 | 393 |
| Alvarez [179] | 2008 | 2006-2007 | Spain | Very high | High | 14 | 384 | 67 | 1.85 W | 38.9 N | ELISA ⁂ | 2416 | 8 |
| de Paschale [180] | 2008 | 2004-2005 | Italy | Very high | high | 12 | 1129 | 77 | 8.90 E | 45.59 N | ELISA and IgG Avidity ⁋ | 3426 | 10 |
| Ocak [181] | 2007 | 2004-2006 | Turkey | High | Upper Middle | 18 | 591 | 57 | 36.34 E | 36.40 N | ELISA # | 1652 | 9 |
| Nowakowska [182] | 2006 | 1998-2003 | Poland | Very high | High | 7 | 676 | 80 | 19.45 E | 51.75 N | ELISA ⁂ | 4916 | 25 |
| Nash [183] | 2005 | 1999-2001 | United Kingdom | Very high | High | 10 | 677 | 84 | 0.52 E | 51.27 N | ELISA and IgG Avidity ⁋ | 1897 | 2 |
| Ertug [184] | 2005 | 2004 | Turkey | High | Upper Middle | 12 | 950 | 62 | 27.50 E | 37.5 N | ELISA and IgG Avidity ⁋ | 389 | 0 |
| Logar [185] | 2005 | 1999-2004 | Slovenia | Very high | High | 10 | 1290 | 74 | 14.5 E | 46 N | ELISA and IgG Avidity ⁋ | 40081 | 153 |
| Antoniou* [186] | 2004 | 1998-2003 | Greece | Very high | High | 19 | 450 | 63 | 25.14 E | 35.33 N | ELISA ⁂ | 5532 | 185 |
| Harma [187] | 2004 | 2002-2003 | Turkey | High | Upper Middle | 11 | 1300 | 68 | 38.79 E | 37.16 N | ELISA # | 1149 | 35 |
| Muñoz Batet [188] | 2004 | 1999 | Spain | Very high | High | 17 | 612 | 77 | 2.1 E | 42.1 N | ELISA ⁂ | 16362 | 12 |
| Gutiérrez-Zufiaurre [189] | 2004 | 2001-2004 | Spain | Very high | High | 12 | 408 | 73 | 5.4 W | 40.5 N | ELISA and IgG Avidity ⁋ | 2929 | 1 |
| Ricci* [190] | 2003 | 1996-2000 | Italy | Very high | High | 15 | 798 | 68 | 12.4 E | 41.9 N | ELISA ⁂ | 8061 | 188 |
| Logar [191] | 2002 | 1996-1999 | Slovenia | Very high | Upper Middle | 10 | 1290 | 81 | 14.3 E | 46 N | ELISA and IgG Avidity ⁋ | 21270 | 132 |
| Niemiec* [192] | 2002 | 2000 | Poland | Very high | High | 7 | 501 | 79 | 20 E | 52.1 N | ELISA ⁂ | 2016 | 5 |
| Vlaspolder [193] | 2001 | 1999 | Netherlands | Very high | High | 10 | 804 | 84 | 4.89 E | 52.37 N | ELISA and IgG Avidity ⁋ | 500 | 3 |
| Evengard* [194] | 2001 | 1997-1998 | Sweden | Very high | High | 7 | 539 | 75 | 18.4 E | 59.19 N | ELISA ⁂ | 40978 | 12 |
| Munoz* [195] | 2000 | 1995-1998 | Spain | Very high | High | 16 | 612 | 74 | 2.1 E | 41.3 N | ELISA ⁂ | 3547 | 26 |
| Sukthana [151] | 1999 | 1997-1998 | Austria | Very high | High | 12 | 700 | 73 | 16.37 E | 48.20 N | ISAGA # | 300 | 19 |
| Hejlicek* [196] | 1999 | 1984-1986 | Czech Republic | Very high | High | 8.3 | 730 | 78 | 14.4 E | 48.9 N | SFT, CFT ⁂ | 1409 | 20 |
| Evengard* [197] | 1999 | 1992-1993 | Sweden | Very high | High | 7 | 539 | 75 | 18.4 E | 59.19 N | ELISA ⁂ | 3,094 | 4 |
| Allain [198] | 1998 | 1992 | U.K. | Very high | High | 10 | 700 | 73 | 1.29 E | 52.63 N | EIA # | 13328 | 36 |
| Stojanovic [199] | 1998 | 1989-1993 | Serbia | Very high | Upper Middle | 12 | 630 | 71 | 22.4 E | 44.1 N | ELISA # | 2778 | 17 |
| Jenum* [200] | 1998 | 1992-1994 | Norway | Very high | High | 6 | 740 | 74 | 10.7 E | 59.9 N | ISAGA ⁂ | 32033 | 47 |
| Szenasi [201] | 1997 | 1987-1994 | Hungary | Very high | High | 11 | 518 | 75 | 21.1 E | 46.2 N | ELISA ⁂ | 17735 | 78 |
| Buffolano [202] | 1996 | 1991-1994 | Italy | Very high | high | 17 | 313 | 72 | 14.26 E | 40.85 N | ELISA # | 3518 | 42 |
| Leone [203] | 1996 | 1993-1994 | Italy | Very high | High | 15 | 798 | 68 | 12.4 E | 41.9 N | ELISA # | 1668 | 10 |
| Lappalainen* [204] | 1995 | 1988-1989 | Finland | Very high | High | 5 | 650 | 80 | 24.9 E | 60.1 N | ELISA and IgG Avidity ⁋ | 16733 | 25 |
| Valcavi [205] | 1995 | 1987-1991 | Italy | Very high | high | 13 | 842 | 75 | 10.32 E | 44.80 N | ELISA ⁂ | 3602 | 5 |
| Lebech [206] | 1995 | 1990-1994 | Denmark | Very high | High | 9 | 621 | 85 | 12.3 E | 55.4 N | ELISA ⁂ | 5402 | 35 |
| Zadik [207] | 1995 | 1989-1992 | United Kingdom | Very high | High | 9.6 | 747 | 84 | 1.4 W | 53.3 N | LAT ⁂ | 1621 | 1 |
| Zuber* [208] | 1995 | 1990-1991 | Switzerland | Very high | High | 10 | 934 | 74 | 6.1 E | 46.2 N | ELISA ⁂ | 5221 | 115 |
| Logar* [209] | 1995 | 1981-1984 | Slovenia | Very high | High | 10 | 1290 | 74 | 14.5 E | 46 N | ELISA, IFA ⁂ | 20953 | 69 |
| Krausse [210] | 1993 | 1986-1990 | Germany | Very high | High | 9 | 570 | 78 | 13.4 E | 52.5 N | ELISA ⁂ | 4355 | 11 |
| Henri* [211] | 1992 | 1966-1987 | Belgium | Very high | High | 10 | 785 | 79 | 4.3 E | 50.8 N | ELISA ⁂ | 20901 | 251 |
| Jaqueti [212] | 1991 | 1987-1989 | Spain | Very high | High | 14 | 450 | 55 | 3.7 W | 40.4 N | ELISA # | 1221 | 15 |
| Ahlfors [213] | 1989 | 1982-1983 | Sweden | Very high | High | 8 | 612 | 92 | 13 E | 55.6 N | ELISA ⁂ | 4351 | 12 |
| Conyn-van Spaendonck* [214] | 1989 | 1986-1987 | Netherlands | Very high | High | 9 | 806 | 87 | 5.2 E | 52.1 N | ELISA ⁂ | 28049 | 55 |
| Foulon* [215] | 1988 | 1979-1986 | Belgium | Very high | High | 10 | 785 | 79 | 4.3 E | 50.8 N | IHA, IFA ⁂ | 6549 | 35 |
| Jeannel* [216] | 1988 | 1981-1983 | France | Very high | High | 11 | 637 | 76 | 2.3 E | 48.8 N | IFAT ⁂ | 2216 | 35 |
| Joss [217] | 1988 | 1980-1986 | Scotland | Very high | High | 8 | 706 | 81 | 3.1 W | 55.9 N | ELISA ⁂ | 4548 | 10 |

|  |  |  |  |  |  |  |  |  |  |  |  |  |  |
| --- | --- | --- | --- | --- | --- | --- | --- | --- | --- | --- | --- | --- | --- |

**Abbreviations:** ATI, acute *Toxoplasma* infection; CMIA, chemiluminescent microparticle immunological assay; EIAs, enzyme immunoassays; ELISA, enzyme-linked immunosorbent assay; ELFA, enzyme-linked fluorescent assay technique; IAT, immunosorbent agglutination test; IFA, immunofluorescence assay; LAT, latex agglutination test; LFIA, Lateral flow chromatographic immunoassay; MEIA, microparticle enzyme immunoassay; SFT, Sabin-Feldman test; Temp, temperature.

* Prospective cohort studies (in these studies sample collection was done at least in two trimesters). All the other studies were cross sectional.

** Sources for income (https://datahelpdesk.worldbank.org/knowledgebase/articles/906519-world-bank-country-and-lending-groups) and HDI levels (http://hdr.undp.org/en/composite/HDI) were from the World Bank Group and the United Nations Development Program.

# In these studies the diagnosis of acute *Toxoplasma* infection was based on positive IgG and IgM ELISA

⁋ In these studies the diagnosis of acute *Toxoplasma* infection was based on positive IgG and IgM ELISA and low IgG avidity

⁂ In these studies the diagnosis of acute *Toxoplasma* infection was based on serocoversion from IgG negative status to IgG positive status

**Referencs**

1. Eshratkhah Mohammadnejad A, Eslami G, Shamsi F, Pirnejad A, Samie A, Safabakhsh J, et al. Prevalence of Food-Borne *Toxoplasma* in Pregnant Women Population of Urmia, Iran. J Food Qual Hazards Control. 2018; 5(1):17–23.

2. Fallahizadeh S, Jelowdar A, Kazemi F, Cheraghian B. Seroprevalence of Anti-*Toxoplasma* IgG and IgM among Pregnant Women of Shush County, Southwest of Iran. Int J Infect. 2018; 5(1):e66810

3. Rehman F, Ahmad R, Jan SS. Prevalence of Abortion among *Toxoplasma gondii* Seropositive Pregnant Women in Community Hospital of Mardan. J Saidu Med College. 2018; 8(1);6–9.

4. Sardarian K, Maghsood AH, Farimani M, Hajiloii M, Saidijam M, Rezaeepoor M, et al. Evaluation of *Toxoplasma gondii* B1 gene in Placental Tissues of Pregnant Women with Acute Toxoplasmosis. Adv Biomed Res. 2018; 7:119.

5. Naghili B, Abbasalizadeh S, Tabrizi S, Rajaii M, Akramiyan M, Alikhah H, et al. Comparison of IIF, ELISA and IgG avidity tests for the detection of anti-*Toxoplasma* antibodies in single serum sample from pregnant women. Infez Med. 2017; 25:50–6.

6. Alanazi FI, Hassan TM, Alanazi WM. Seroprevalence of *Toxoplasma gondii* among pregnant Saudi woman in Arar, Northern Borders Province, Saudi Arabia. Kasr Al Ainy Med J. 2017; 23(2):104.

7. Laboudi M, Sadak A. Serodiagnosis of Toxoplasmosis: the effect of measurement of IgG avidity in Pregnant Women in Rabat in Morocco. Acta Trop. 2017; 172:139–42.

8. Mahdy MA, Alareqi LM, Abdul-Ghani R, Al-Eryani SM, Al-Mikhlafy AA, Al-Mekhlafi AM, et al. A community-based survey of *Toxoplasma gondii* infection among pregnant women in rural areas of Taiz governorate, Yemen: the risk of waterborne transmission. Infect Dis Poverty. 2017; 6(1):26.

9. Nahouli H, El Arnaout N, Chalhoub E, Anastadiadis E, El Hajj H. Seroprevalence of Anti-*Toxoplasma gondii* Antibodies Among Lebanese Pregnant Women. Vector-Borne Zoonotic Dis. 2017; 17(12):785–90.

10. Norouzi LY, Sarkari B, Asgari Q, Khabisi SA. Molecular Evaluation and Seroprevalence of Toxoplasmosis in Pregnant Women in Fars province, Southern Iran. Ann Med Health Sci Res. 2017; 7(1):16–9.

11. Shieh M, Didehdar M, Hajihossein R, Ahmadi F, Eslamirad Z. Toxoplasmosis: Seroprevalence in pregnant women, and serological and molecular screening in neonatal umbilical cord blood. Acta Trop. 2017; 174:38–44.

12. Ahmadpour GR, Ezatpour B, Hadighi R, Oormazdi H, Akhlaghi L, Tabatabaei F, et al. Seroepidemiology of *Toxoplasma gondii* infection in pregnant women in west Iran: determined by ELISA and PCR analysis. J Parasitic Dis. 2017; 41(1):237–42.

13. Garedaghi Y, Firozivand Y. Assessment of Pregnant Women Toxoplasmosis by ELISA Method in Miandoab City, Iran. Int J Womens Health Reprod Sci. 2017; 5(1):72–5.

14. Shirdel S, Sharbatkhori M, Pagheh AS, Dadimoghadam Y, Soosaraie M, Gholami S. Seroepidemiology of *Toxoplasma gondii* Infection in Pregnant Women and Risk Factors of the Disease in Golestan Province, Iran. J Maz Univ Med Sci. 2017; 27(152):63–71.

15. Akhlaghi L, Tabatabaie F, Hadighi R, Maleki F, Hajialiani F, Dayer MS, et al. Diagnosis of acute toxoplasmosis in pregnant women referred to therapeutic centers of Alborz Province (Iran) using immunoglobulin G avidity ELISA technique. Asian Pacific J Trop Dis. 2016; 6(11):864–7.

16. Al-Eryani SM, Al-Mekhlafi AM, Al-Shibani LA, Mahdy MM, Azazy AA. *Toxoplasma gondii* infection among pregnant women in Yemen: Factors associated with high seroprevalence. J Infect Dev Ctries. 2016; 10(06):667–72.

17. Alghamdi J, Elamin MH, Alhabib S. Prevalence and genotyping of *Toxoplasma gondii* among Saudi pregnant women in Saudi Arabia. Saudi Pharm J. 2016; 24(6):645–51.

18. Mohaghegh MA, Kalani H, Hashemi M, Hashemi S, Yazdnezhad SK, Hejazi SH, et al. Toxoplasmosis-related risk factors in pregnant women in the North Khorasan province, Iran. Health Sci. 2016; 5(8):370–4.

19. Mohamed K, Bahathiq A, Degnah N, Basuni S, Al Malki A, Babalghith A. Detection of *Toxoplasma gondii* infection and associated risk factors among pregnant women in Makkah Al Mukarramah, Saudi Arabia. Asian Pacific J Trop Dis. 2016; 6(2):113–9.

20. Rostamzadeh Khameneh Z, Hanifian H, Rostamzadeh A. Seroprevalence of Toxoplasmosis in Pregnant Women in Urmia, Iran. Int J Enteric Pathog. 2016; 4(2):1–3.

21. Bassiouny HK, Soliman NK, El Tawab S, Eassa SM, Eissa A. Sero-prevalence and risk factors associated with *Toxoplasma gondii* infection among pregnant women in Alexandria, Egypt. Int J Reprodu Contracep Obstetr Gynecol. 2016; 5(12):4220–7.

22. Imam NF, Esra'a A, Attia AA. Seroprevalence of *Toxoplasma gondii* among pregnant women in Almadinah Almunawwarah KSA. J Taibah Unive Med Sci. 2016; 11(3):255–9.

23. Tabatabaie F, Mafi M, Golestani M, Shahmohammad N, Mafi H, Maleki F. Seroprevalence of and risk factors for *Toxoplasma gondii* among pregnant women in abyek township of Qazvin province, Iran (2013). Asian J Pharm Clin Res. 2015; 8:1–3.

24. Elsafi SH, AL-Mutairi WF, Al-Jubran KM, Abu Hassan MM, Al Zahrani EM. Toxoplasmosis seroprevalence in relation to knowledge and practice among pregnant women in Dhahran, Saudi Arabia. Pathog Glob Health. 2015; 109(8):377–82.

25. Kamal AM, Ahmed AK, Abdellatif MZ, Tawfik M, Hassan EE. Seropositivity of toxoplasmosis in pregnant women by ELISA at Minia University Hospital, Egypt. Korean J Parasitol. 2015; 53(5):605–10.

26. Abdel-Raouff M, Elbasheir MM. Sero-prevalence of *Toxoplasma gondii* infection among pregnant women attending antenatal clinics in Khartoum and Omdurman Maternity Hospitals, Sudan. J Coastal Life Med. 2014; 2(6):496–9.

27. Sharbatkhori M, Moghaddam YD, Pagheh AS, Mohammadi R, Mofidi HH, Shojaee S. Seroprevalence of *Toxoplasma gondii* infections in pregnant women in Gorgan city, Golestan Province, Northern Iran-2012. Iran J Parasitol. 2014; 9(2):181–7.

28. Yad Yad MJ, Jomehzadeh N, Sameri MJ, Noorshahi N. Seroprevalence of Anti-*Toxoplasma gondii* antibodies among pregnant woman in South Khuzestan, Iran. Jundishapur J Microbiol. 2014; 7(5):e9998.

29. Aqeely H, El-Gayar EK, Perveen Khan D, Najmi A, Alvi A, Bani I, et al. Seroepidemiology of *Toxoplasma gondii* amongst pregnant women in Jazan Province, Saudi Arabia. J Trop Med. 2014; 2014; 913950.

30. Ghasemloo H, Ghomashlooyan M, Hooshyar H. Seroprevalence of *Toxoplasma gondii* infection among pregnant women admitted at Shahid Akbar Abadi hospital, Tehran, Iran, 2010-2013. J Med Microbiol Infect Dis. 2014; 2(1):16–8.

31. Ahmed HA, Shafik SM, Ali ME, Elghamry ST, Ahmed AA. Molecular detection of *Toxoplasma gondii* DNA in milk and risk factors analysis of seroprevalence in pregnant women at Sharkia, Egypt. Vet World. 2014; 7(8);594–600.

32. Ashankyty IM. Seroprevalence of *Toxoplasma gondii* among pregnant women visiting maternity hospital in Hail, KSA. Life Sci J. 2014; 11(8):355e9.

33. Almushait MA, Dajem SMB, Elsherbiny NM, Eskandar MA, Al Azraqi TA, Makhlouf LM. Seroprevalence and risk factors of *Toxoplasma gondii* infection among pregnant women in south western, Saudi Arabia. J Parasitic Dis. 2014; 38(1):4-10.

34. Ben RA, Siala E, Bouafsoun A, Maatoug R, Souissi O, Aoun K, et al. Toxoplasmosis mother-to-child screening: study of cases followed in the Pasteur Institute of Tunis (2007-2010). Bull Soc Pathol Exot. 2013; 106(2):108-12.

35. Akhlaghi L, Shirbazou S, Maleki F, Keyghobadi A, Tabaraei Y, Tabatabaie F. Seroepidemiology of *Toxoplasma* infection in pregnant women in Qom province, Iran (2010). Life Sci J. 2013; 10(7s):322–5.

36. Babaie J, Amiri S, Mostafavi E, Hassan N, Lotfi P, Rastaghi ARE, et al. Seroprevalence and risk factors for *Toxoplasma gondii* infection among pregnant women in Northeast Iran. Clin Vaccine Immunol. 2013; 20(11):1771–3.

37. El Deeb HK, Salah-Eldin H, Khodeer S, Allah AA. Prevalence of *Toxoplasma gondii* infection in antenatal population in Menoufia governorate, Egypt. Acta Trop. 2012; 124(3):185–91.

38. Elamin MH, Al-Olayan EM, Omer SA, Alagaili AN, Mohammed OB. Molecular detection and prevalence of *Toxoplasma gondii* in pregnant women in Sudan. Afr J Microbiol Res. 2012; 6(2):308–11.

39. Hajsoleimani F, Ataeian A, Nourian A, Mazloomzadeh S. Seroprevalence of *Toxoplasma gondii* in pregnant women and bioassay of IgM positive cases in Zanjan, Northwest of Iran. Iran J Parasitol. 2012; 7(2):82–6.

40. Bin-Dajem SM, Almushaitb MA. Detection of *Toxoplasma gondii* DNA by PCR in blood samples collected from pregnant Saudi women from the Aseer region, Saudi Arabia. Ann Saudi Med. 2012; 32(5):507–12.

41. Almogren A. Antenatal screening for *Toxoplasma gondii* infection at a tertiary care hospital in Riyadh, Saudi Arabia. Ann Saudi Med. 2011; 31(6):569–72.

42. Mohammad HA, Amin T, Balaha M, Moghannum MA. Toxoplasmosis among the pregnant women attending a Saudi maternity hospital: seroprevalence and possible risk factors. Ann Trop Med Parasitol. 2010; 104(6):493–504.

43. Al-Nahari AM, Al-Tamimi A-HS. Seroprevalence Of Anti *Toxoplasma gondii* IgG and IgM Among Pregnant Women in Sana’a Capital and Capital Trusteeship. Sci J King Faisal Univ. 2010; 11(2):1431.

44. Parvizpour F, Hajighasemlo S, Hasani S, Olfati L, Bahmani A, Hoseini F, et al. Toxoplasmosis infection in the pregnant women in the first half of pregnancy, in Kamyaran in 2008. Sci J Kurdistan Unive Med Sci. 2010; 15(1):72–8.

45. Iqbal J, Khalid N. Detection of acute *Toxoplasma gondii* infection in early pregnancy by IgG avidity and PCR analysis. J Med Microbiol. 2007; 56(11):1495–9.

46. Saeedi M, Veghari GR, Marjani A. Seroepidemiologic evaluation of anti-*Toxoplasma* antibodies among women in north of Iran. Pak J Biol Sci. 2007; 10(14):2359–62.

47. Al-Harthi SA, Jamjoom MB, Ghazi HO. Seroprevalence of *Toxoplasma gondii* among pregnant women in Makkah, Saudi Arabia. Umm Al-Qura Univ J Science Med Eng. 2006; 18:217–27.

48. Elnahas A, Gerais AS, Elbashir MI, Eldien ES, Adam I. Toxoplasmosis in pregnant Sudanese women. Saudi Med J. 2003; 24(8):868–70.

49. Noorbakhsh S, Mamishi S, Rimaz S, Monavari S. Toxoplasmosis in primiparus pregnant women and their neonates. Iran J Public Health. 2002; 31(1-2):51–4.

50. Al-Mulhim AA, Al-Qurashi AM. Sero-prevalence of toxoplasmosis in pregnant mothers and new born infants in eastern province, Saudi Arabia. J Family Community Med. 2001; 8(1):45–8.

51. Dar F, Alkarmi T, Uduman S, Abdulrazzaq Y, Grundsell H, Hughes P. Gestational and neonatal toxoplasmosis: regional seroprevalence in the United Arab Emirates. Eur J Epidemiol. 1997; 13(5):567–71.

52. El-Nawawy A, Soliman AT, El Azzouni O, Amer E-S, Karim MA, Demian S, et al. Maternal and neonatal prevalence of *Toxoplasma* and cytomegalovirus (CMV) antibodies and hepatitis-B antigens in an Egyptian rural area. J Trop Pediatr. 1996; 42(3):154–7.

53. Franklin D, Dror Z, Nishri Z. The prevalence and incidence of *Toxoplasma* antibodies in pregnant women. Israel J Med Sci. 1993; 29(5):285–6.

54. Paul E, Kiwelu I, Mmbaga B, Nazareth R, Sabuni E, Maro A, et al. *Toxoplasma gondii* seroprevalence among pregnant women attending antenatal clinic in Northern Tanzania. Trop Med Health. 2018; 46(1):39.

55. Berredjem H, Aouras H, Benlaifa M, Becheker I, Djebar MR. Contribution of IgG avidity and PCR for the early diagnosis of toxoplasmosis in pregnant women from the North-Eastern region of Algeria. Afr Health Sci. 2017; 17(3):647–56.

56. Murebwayire E, Njanaake K, Ngabonziza JCS, Jaoko W, Njunwa KJ. Seroprevalence and risk factors of *Toxoplasma gondii* infection among pregnant women attending antenatal care in Kigali, Rwanda. Tanzania J Health Res. 2017; 19(1).

57. Yohanes T, Zerdo Z, Chufamo N, Abossie A. Seroprevalence and Associated Factors of *Toxoplasma gondii* Infection among Pregnant Women Attending in Antenatal Clinic of Arba Minch Hospital, South Ethiopia: Cross Sectional Study. Transl Biomed. 2017; 8:1.

58. Bamba S, Cissé M, Sangaré I, Zida A, Ouattara S, Guiguemdé RT. Seroprevalence and risk factors of *Toxoplasma gondii* infection in pregnant women from Bobo Dioulasso, Burkina Faso. BMC Infect Dis. 2017; 17(1):482.

59. Frimpong C, Makasa M, Sitali L, Michelo C. Seroprevalence and determinants of toxoplasmosis in pregnant women attending antenatal clinic at the university teaching hospital, Lusaka, Zambia. BMC Infect Dis. 2017; 17(1):10.

60. Völker F, Cooper P, Bader O, Uy A, Zimmermann O, Lugert R, et al. Prevalence of pregnancy-relevant infections in a rural setting of Ghana. BMC Pregnancy Childbirth. 2017; 17(1):172.

61. Lobo M, Patrocinio G, Sevivas T, De Sousa B, Matos O. Portugal and Angola: similarities and differences in *Toxoplasma gondii* seroprevalence and risk factors in pregnant women. Epidemiol Infect. 2017; 145(1):30–40.

62. Abamecha F, Awel H. Seroprevalence and risk factors of *Toxoplasma gondii* infection in pregnant women following antenatal care at Mizan Aman General Hospital, Bench Maji Zone (BMZ), Ethiopia. BMC Infect Dis. 2016; 16(1):460.

63. Ayi I, Sowah AO-K, Blay EA, Suzuki T, Ohta N, Ayeh-Kumi PF. *Toxoplasma gondii* infections among pregnant women, children and HIV-seropositive persons in Accra, Ghana. Trop Med Health. 2016; 44(1):17.

64. Nasir IA, Aderinsayo AH, Mele HU, Aliyu MM. Prevalence and associated risk factors of *Toxoplasma gondii* antibodies among pregnant women attending Maiduguri teaching hospital, Nigeria. J Med Sci. 2015; 15(3):147.

65. Messerer L, Bouzbid S, Gourbdji E, Mansouri R, Bachi F. Séroprévalence de la toxoplasmose chez les femmes enceintes dans la wilaya d’Annaba, Algérie. Rev Épidémiol Santé Publique. 2014; 62(2):160–5.

66. Doudou Y, Renaud P, Jacqueline F, Hypolite S, Hypolite M, Patrick M, et al. Toxoplasmosis among pregnant women: high seroprevalence and risk factors in Kinshasa, Democratic Republic of Congo. Asian Pacific J Trop Biomed. 2014; 4(1):69–74.

67. De Paschale M, Ceriani C, Cerulli T, Cagnin D, Cavallari S, Cianflone A, et al. Antenatal screening for *Toxoplasma gondii*, Cytomegalovirus, rubella and Treponema pallidum infections in northern Benin. Trop Med Int Health. 2014; 19(6):743–6.

68. Mwambe B, Mshana SE, Kidenya BR, Massinde AN, Mazigo HD, Michael D, et al. Sero-prevalence and factors associated with *Toxoplasma gondii* infection among pregnant women attending antenatal care in Mwanza, Tanzania. Parasite Vectors. 2013; 6(1):222.

69. Zemene E, Yewhalaw D, Abera S, Belay T, Samuel A, Zeynudin A. Seroprevalence of *Toxoplasma gondii* and associated risk factors among pregnant women in Jimma town, Southwestern Ethiopia. BMC Infect Dis. 2012; 12(1):337.

70. Linguissi LSG, Nagalo BM, Bisseye C, Kagoné TS, Sanou M, Tao I, et al. Seroprevalence of toxoplasmosis and rubella in pregnant women attending antenatal private clinic at Ouagadougou, Burkina Faso. Asian Pacific J Trop Med. 2012; 5(10):810–3.

71. Njunda AL, Assob JC, Nsagha DS, Kamga HL, Nde PF, Yugah VC. Seroprevalence of *Toxoplasma gondii* infection among pregnant women in Cameroon. J Public Health Afr. 2011; 2(2):e24.

72. Ndiaye D, Sene P, Ndiaye M, Faye B, Ndiaye J, Ndir O. Update on toxoplasmosis prevalence based on serological tests in pregnant women in Dakar, Senegal from 2002 to 2006. Med Trop. 2011; 71(1):101–2.

73. Sitoe SPBL, Rafael B, Meireles LR, Andrade Jr HFd, Thompson R. Preliminary report of HIV and *Toxoplasma gondii* occurrence in pregnant women from Mozambique. Rev Instit Med Trop São Paulo. 2010; 52(6):291–5.

74. Mickoto B, Akue J, Bisvigou U, Tsonga SM, Nkoghe D. Serological study on toxoplasmosis among pregnant women from Franceville, Gabon. Bull Soc Pathol Exot. 2010; 103(1):41–3.

75. Ayi I, Edu S, Apea-Kubi K, Boamah D, Bosompem K, Edoh D. Sero-epidemiology of toxoplasmosis amongst pregnant women in the greater Accra region of Ghana. Ghana Med J. 2009; 43(3);107–14.

76. Ishaku BS, Ajogi I, Umoh JU, Lawal I, Randawa AJ. Seroprevalence and Risk Factors for *Toxoplasma gondii* Infection among Antenatal Women in Zaria, Nigeria. Res J Med Med Sci. 2009; 4(2):483–8.

77. Ndiaye D, Ndiaye A, Sene P, Ndiaye J, Faye B, Ndir O. Evaluation of serological tests of toxoplasmosis in pregnant women realized at the laboratory of parasitology and mycology of Le Dantec Teaching Hospital in 2002. Dakar Med. 2007; 52(1):58–61.

78. Simpore J, Savadogo A, Ilboudo D, Nadambega MC, Esposito M, Yara J, et al. *Toxoplasma gondii*, HCV, and HBV seroprevalence and co‐infection among HIV‐positive and‐negative pregnant women in Burkina Faso. J Med Virol. 2006; 78(6):730–3.

79. Diallo S, Ndir O, Dieng Y, Leye A, Dieng T, Bah I, et al. Seroprevalence of toxoplasmosis in Dakar (Senegal) in 1993: study of women in their reproductive years. Sante (Montrouge, France). 1996; 6(2):102–6.

80. Rodier M, Berthonneau J, Bourgoin A, Giraudeau G, Agius G, Burucoa C, et al. Seroprevalences of *Toxoplasma*, malaria, rubella, cytomegalovirus, HIV and treponemal infections among pregnant women in Cotonou, Republic of Benin. Acta Trop. 1995; 59(4):271–7.

81. Jiang R-L, Ma L-H, Ma Z-R, Hou G, Zhao Q, Wu X. Seroprevalence and associated risk factors of *Toxoplasma gondii* among Manchu pregnant women in northeastern China. Microb Pathog. 2018; 123:398–401.

82. Zhou J, Tao L. Seroprevalence and risk factors of *Toxoplasma gondii* infection among pregnant women in Wuxi region. Chinese J Schistosomiasis Control. 2015; 27(6):604–7.

83. Cong W, Dong X-Y, Meng Q-F, Zhou N, Wang X-Y, Huang S-Y, et al. *Toxoplasma gondii* infection in pregnant women: a seroprevalence and case-control study in Eastern China. BioMed Res Int. 2015; 2015:170278.

84. Hung C-S, Su H-W, Lee Y-L, Weng H-W, Wang Y-C, Naito T, et al. Seroprevalence, seroconversion, and risk factors for toxoplasmosis among pregnant women in Taipei, Taiwan. Jpn J Infect Dis. 2015; 68(4):312–7.

85. Chen Y, Yang Y, Wei S, Song R. Impact of *Toxoplasma gondii* infection on pregnancy outcomes in early pregnant women. Chinese J Schistosomiasis Control. 2014; 26(3):308–10.

86. Andiappan H, Nissapatorn V, Sawangjaroen N, Nyunt MH, Lau Y-L, Khaing SL, et al. Comparative study on *Toxoplasma* infection between Malaysian and Myanmar pregnant women. Parasit Vectors. 2014; 7(1):564.

87. Zhang Y, Song R. Investigation on pregnancy outcomes and risk factors in pregnant women infected with *Toxoplasma gondii*. Chinese J Schistosomiasis Control. 2014; 26(2):221–3.

88. Emelia O, Rahana A, Mohamad Firdaus A, Cheng H, Nursyairah M, Fatinah A, et al. IgG avidity assay: a tool for excluding acute toxoplasmosis in prolonged IgM titer sera from pregnant women. Trop Biomed. 2014; 31(4):633–40.

89. Hua H, Tang F, Liu Y, You L, Dong M, Chen Y, et al. Survey of *Toxoplasma gondii* infection among pregnant women in Jiangsu Province, China. Chinese J Schistosomiasis Control. 2013; 25(1):56–8.

90. Sakikawa M, Noda S, Hanaoka M, Nakayama H, Hojo S, Kakinoki S, et al. Anti-*Toxoplasma* antibody prevalence, primary infection rate, and risk factors in a study of toxoplasmosis in 4,466 pregnant women in Japan. Clin Vaccine Immunol. 2012; 19(3):365–7.

91. Chou C, Lin L, Chen K, Lai S. Flowcytomix analysis for *Toxoplasma gondii* infection in pregnant women in central Taiwan. J Obstetr Gynaecol. 2011; 31(5):375–9.

92. Xia W, Zhang X, Chen X. Investigation of different pregnant results of pregnant women infected with *Toxoplasma gondii* in Nanjing region. Chinese J Schistosomiasis Control. 2011; 23(2):183–6.

93. Huang H, Liang L-q, Huang J-c, Zhang G-x, Zhu T-y. Serological investigation of *Toxoplasma* infection among Renshou pregnant womem. Chinese J Health Lab Technol. 2010; 5:118.

94. Liu Q, Wei F, Gao S, Jiang L, Lian H, Yuan B, et al. *Toxoplasma gondii* infection in pregnant women in China. Trans R Soc Trop Med Hyg. 2009; 103(2):162–6.

95. Lin Y-L, Liao Y-S, Liao L-R, Chen F-N, Kuo H-M, He S. Seroprevalence and sources of *Toxoplasma* infection among indigenous and immigrant pregnant women in Taiwan. Parasitol Res. 2008; 103(1):67–74.

96. Hu I-J, Chen P-C, Su F-C, Hsieh C-J, Jeng S-F, Liao H-F, et al. Perinatal toxoplasmosis, northern Taiwan. Emerg Infect Dis. 2006; 12(9):1460.

97. Song K-J, Shin J-C, Shin H-J, Nam H-W. Seroprevalence of toxoplasmosis in Korean pregnant women. Korean J Parasitol. 2005; 43(2):69–71.

98. Morris A, Croxson M. Serological evidence of *Toxoplasma gondii* infection among pregnant women in Auckland. New Zealand Med J. 2004; 117(1189).

99. Buchy P, Follezou J, Lien T, An T, Tram L, Tri Da, et al. Serological study of toxoplasmosis in Vietnam in a population of drug users (Ho Chi Minh city) and pregnant women (Nha Trang). Bull Soc Pathol Exot. 2003; 96(1):46–7.

100. Zhang A, Zhang T, Hao Z. A seroepidemic survey on the infection of *Toxoplasma* in pregnant women and its significance to better child-bearing. Zhonghua liu xing bing xue za zhi. 1996; 17(5):278–80.

101. Sun R, Liu Z, Wang D. The prevalence of *Toxoplasma* infection among pregnant women and their newborn infants in Chengdu. Zhonghua liu xing bing xue za zhi. 1995; 16(2):98–100.

102. Walpole I, Hodgen N, Bower C. Congenital toxoplasmosis: a large survey in western Australia. Med J Aust. 1991; 154(11):720–4.

103. Sfameni SF, Skurrie IJ, Gilbert GL. Antenatal screening for congenital infection with rubella, cytomegalovirus and *Toxoplasma*. Aust New Zealand J Obstetr Gynaecol. 1986; 26(4):257–60.

104. Barbosa Costa G, de Oliveira MC, Gadelha SR, Albuquerque GR, Teixeira M, da Silva Raiol MR, et al. Infectious diseases during pregnancy in Brazil: seroprevalence and risk factors. J Infect Dev Ctries. 2018; 12(08):657–65.

105. Avelar MV, Martinez VO, Moura DLd, Barros IA, Primo AAdS, Duarte AO, et al. Association between seroprevalence of IgG anti-*Toxoplasma gondii* and risk factors for infection among pregnant women in Climério de Oliveira Maternity, Salvador, Bahia, Brazil. Rev Instit Med Trop São Paulo. 2017; 59:90.

106. Alvarado-Esquivel C, del Carmen Terrones-Saldívar M, Hernández-Tinoco J, Muñoz-Terrones MDE, Gallegos-González RO, Sánchez-Anguiano LF, et al. Seroepidemiology of *Toxoplasma gondii* in pregnant women in Aguascalientes City, Mexico: a cross-sectional study. BMJ Open. 2016; 6(7):e012409.

107. Dubey JP, Verma SK, Villena I, Aubert D, Geers R, Su C, et al. Toxoplasmosis in the Caribbean islands: literature review, seroprevalence in pregnant women in ten countries, isolation of viable *Toxoplasma gondii* from dogs from St. Kitts, West Indies with report of new *T. gondii* genetic types. Parasitol Res. 2016; 115(4):1627–34.

108. da Silva MG, Vinaud MC, de Castro AM. Prevalence of toxoplasmosis in pregnant women and vertical transmission of *Toxoplasma gondii* in patients from basic units of health from Gurupi, Tocantins, Brazil, from 2012 to 2014. PloS One. 2015; 10(11):e0141700.

109. Quadros RMd, Rocha GCd, Romagna G, Oliveira JPd, Ribeiro DM, Marques SMT. *Toxoplasma gondii* seropositivity and risk factors in pregnant women followed up by the Family Health Strategy. Rev Soc Bras Med Trop. 2015; 48(3):338–42.

110. Rocha ÉMd, Lopes CWG, Ramos RAN, Alves LC. Risk factors for *Toxoplasma gondii* infection among pregnant women from the State of Tocantins, Northern Brazil. Rev Soc Bras Med Trop. 2015; 48(6):773–5.

111. Ferezin RI, Bertolini DA, Demarchi IG. Prevalence of positive sorology for HIV, hepatitis B, toxoplasmosis and rubella in pregnant women from the northwestern region of the state of Paraná. Rev Bras Ginecol Obstetr. 2013; 35(2):66–70.

112. Fonseca AL, Silva RA, Fux B, Madureira AP, Sousa FFd, Margonari C. Epidemiologic aspects of toxoplasmosis and evaluation of its seroprevalence in pregnant women. Rev Soc Brasil Med Trop. 2012; 45(3):357–64.

113. Bittencourt LHFdB, Lopes-Mori FMR, Mitsuka-Breganó R, Valentim-Zabott M, Freire RL, Pinto SB, et al. Seroepidemiology of toxoplasmosis in pregnant women since the implementation of the Surveillance Program of Toxoplasmosis Acquired in Pregnancy and Congenital in the western region of Paraná, Brazil. Rev Bras Ginecol Obstetr. 2012; 34(2):63–8.

114. Dias RCF, Lopes-Mori FMR, Mitsuka-Breganó R, Dias RAF, Tokano DV, Reiche EMV, et al. Factors associated to infection by *Toxoplasma gondii* in pregnant women attended in Basic Health Units in the city of Rolândia, Paraná, Brazil. Rev Instit Med Trop São Paulo. 2011; 53(4):185–91.

115. Sartori AL, Minamisava R, Avelino MM, Martins CA. Prenatal screening for toxoplasmosis and factors associated with seropositivity of pregnant women in Goiânia, Goiás. Rev Bras Ginecol Obstetr. 2011; 33(2):93–8.

116. Sroka S, Bartelheimer N, Winter A, Heukelbach J, Ariza L, Ribeiro H, et al. Prevalence and risk factors of toxoplasmosis among pregnant women in Fortaleza, Northeastern Brazil. Am J Trop Med Hyg. 2010; 83(3):528–33.

117. Vaz RS, Thomaz-Soccol V, Sumikawa E, Guimarães ATB. Serological prevalence of *Toxoplasma gondii* antibodies in pregnant women from Southern Brazil. Parasitol Res. 2010; 106(3):661–5.

118. Alarcón de Noya B, Romero J, Sánchez E, Jesús L, Salinas R, Ortiz L, et al. Despistaje de toxoplasmosis y enfermedad de Chagas en la Consulta Prenatal del Hospital Universitario de Caracas. Rev Obstetr Ginecol Venezuela. 2010; 70(2):75–81.

119. Gonçalves MAdS, Matos CdCBd, Spegiorin LCJF, Oliani DCMV, Oliani AH, Mattos LCd. Seropositivity rates for toxoplasmosis, rubella, syphilis, cytomegalovirus, hepatitis and HIV among pregnant women receiving care at a public health service, São Paulo state, Brazil. Braz J Infect Dis. 2010; 14(6):601–5.

120. Varella IS, Canti IC, Santos BR, Coppini AZ, Argondizzo LC, Tonin C, et al. Prevalence of acute toxoplasmosis infection among 41,112 pregnant women and the mother-to-child transmission rate in a public hospital in South Brazil. Mem Inst Oswaldo Cruz. 2009; 104(2):383–8.

121. Lopes F, Mitsuka-Breganó R, Gonçalves D, Freire R, Karigyo C, Wedy G, et al. Factors associated with seropositivity for anti-*Toxoplasma gondii*antibodies in pregnant women of Londrina, Paraná, Brazil. Mem Inst Oswaldo Cruz. 2009; 104(2):378–82.

122. Madi JM, Souza RdSd, Araújo BFd, Oliveira Filho PFd, Rombaldi RL, Mitchell C, et al. Prevalence of toxoplasmosis, HIV, syphilis and rubella in a population of puerperal women using Whatman 903® filter paper. Braz J Infect Dis. 2010; 14(1):24–9.

123. Alvarado-Esquivel C, Torres-Castorena A, Liesenfeld O, García-López C, Estrada-Martínez S, Sifuentes-Alvarez A, et al. Seroepidemiology of *Toxoplasma gondii* infection in pregnant women in rural Durango, Mexico. J Parasitol. 2009; 95(2):271–4.

124. Barbosa IR, Holanda CMdCX, de Andrade-Neto VF. Toxoplasmosis screening and risk factors amongst pregnant females in Natal, northeastern Brazil. Trans R Soc Trop Med Hyg. 2009; 103(4):377–82.

125. Carral L, Kaufer F, Durlach R, Freuler C, Olejnik P, Nadal M, et al. Multicenter study on the prevention of congenital toxoplasmosis in Buenos Aires. Medicina. 2008; 68(6):417–22.

126. Ramsewak S, Gooding R, Ganta K, Seepersadsingh N, Adesiyun AA. Seroprevalence and risk factors of *Toxoplasma gondii* infection among pregnant women in Trinidad and Tobago. Rev Panam Salud Publica. 2008; 23:164–70.

127. Rosso F, Les JT, Agudelo A, Villalobos C, Chaves JA, Tunubala GA, et al. Prevalence of infection with *Toxoplasma gondii* among pregnant women in Cali, Colombia, South America. Am J Trop Med Hyg. 2008; 78(3):504–8.

128. Ribeiro AC, Mutis MS, Fernandes O. Association of the presence of residual anti-*Toxoplasma gondii* IgM in pregnant women and their respective family groups in Miracema, Northwest Rio de Janeiro, Brazil. Mem Inst Oswaldo Cruz. 2008; 103(6):591–4.

129. Porto AMF, Amorim MMRd, Coelho ICN, Santos LC. Serologic profile of toxoplasmosis in pregnant women attended at a teaching-hospital in Recife. Rev Assoc Med Bras. 2008; 54(3):242–8.

130. Castilho-Pelloso MP, Falavigna DLM, Falavigna-Guilherme AL. Suspected acute toxoplasmosis in pregnant women. Rev Saúde Publica. 2007; 41(1):27–34.

131. Alvarado-Esquivel C, Sifuentes-Álvarez A, Narro-Duarte SG, Estrada-Martínez S, Díaz-García JH, Liesenfeld O, et al. Seroepidemiology of *Toxoplasma gondii* infection in pregnant women in a public hospital in northern Mexico. BMC Infect Dis. 2006; 6(1):113.

132. Spalding SM, Amendoeira MRR, Klein CH, Ribeiro LC. Serological screening and toxoplasmosis exposure factors among pregnant women in South of Brazil. Rev Soc Bras Med Trop. 2005; 38(2):173–7.

133. Nóbrega OdT, Karnikowski MGdO. An estimation of the frequency of gestational toxoplasmosis in the Brazilian Federal District. Rev Soc Bras Med Trop. 2005; 38(4):358–60.

134. Avelino MM, Campos Jr D, de Parada JdCB, de Castro AM. Pregnancy as a risk factor for acute toxoplasmosis seroconversion. Eur J Obstetr Gynecol Reprod Biol. 2003; 108(1):19–24.

135. Gomez-Marin JE, Montoya-de-Londono MT, Castano-Osorio JC. A maternal screening program for congenital toxoplasmosis in Quindio, Colombia and application of mathematical models to estimate incidences using age-stratified data. Am J Trop Med Hyg. 1997; 57(2):180–6.

136. Gonzalez-Morales T, Bacallo-Gallestey J, Garcia-Santana C, Molina-Garcia J. Prevalence of *Toxoplasma gondii* antibodies in a population of pregnant women in Cuba. Gaceta Med Mexico. 1995; 131(5-6):499–503.

137. Naheen C, Tarafder S, Khan S. *Toxoplasma gondii* Seropositivity and its Possible Associated Risk Factors among Pregnant Women Attending Antenatal Clinic in a Tertiary Care Hospital of Dhaka, Bangladesh. Mymensingh Medical J. 2018; 27(2):336–43.

138. Stephen S, Anitharaj V, Ghose S, Pradeep J. Seroprevalence of *Toxoplasma gondii* in Healthy Pregnant Women of Puducherry. J Krishna Inst Med Sci Univ. 2017; 6(4):134–6.

139. van Enter BJ, Lau Y-L, Ling CL, Watthanaworawit W, Sukthana Y, Lee W-C, et al. Seroprevalence of *Toxoplasma gondii* Infection in Refugee and Migrant Pregnant Women along the Thailand–Myanmar Border. Am J Trop Med Hyg. 2017; 97(1):232–5.

140. Iddawela D, Vithana SMP, Ratnayake C. Seroprevalence of toxoplasmosis and risk factors of *Toxoplasma gondii* infection among pregnant women in Sri Lanka: a cross sectional study. BMC Public Health. 2017; 17(1):930.

141. Chandrasena N, Herath R, Rupasinghe N, Samarasinghe B, Samaranayake H, Kastuririratne A, et al. Toxoplasmosis awareness, seroprevalence and risk behavior among pregnant women in the Gampaha district, Sri Lanka. Pathog Glob Health. 2016; 110(2):62–7.

142. Amar OAO, Bajaj HK, Peter JK, Masih H. Toxoplasmosis Prevalence in Pregnant Women of Plain Gangetic Region, Allahabad. J Pure App Microbiol. 2015; 9(2):1611–9.

143. Singh S, Munawwar A, Rao S, Mehta S, Hazarika NK. Serologic prevalence of *Toxoplasma gondii* in Indian women of child bearing age and effects of social and environmental factors. PLoS Negl Trop Dis. 2014; 8(3):e2737.

144. Andiappan H, Nissapatorn V, Sawangjaroen N, Chemoh W, Lau YL, Kumar T, et al. *Toxoplasma* infection in pregnant women: a current status in Songklanagarind hospital, southern Thailand. Parasit Vectors. 2014; 7(1):239.

145. Malarvizhi A, Viswanathan T, Lavanya V, Moorthy K. Seroprevalence of *Toxoplasma gondii* in pregnant women. J Public Health Epidemiol. 2012; 4(6):170–7.

146. Nissapatorn V, Suwanrath C, Sawangjaroen N, Ling LY, Chandeying V. Toxoplasmosis-serological evidence and associated risk factors among pregnant women in southern Thailand. American J Trop Med Hyg. 2011; 85(2):243–7.

147. Khurana S, Bagga R, Aggarwal A, Lyngdoh V, Diddi K, Malla N. Serological screening for antenatal *Toxoplasma* infection in India. Indian J Med Microbiol. 2010; 28(2):143.

148. Singh S, Pandit AJ. Incidence and prevalence of toxoplasmosis in Indian pregnant women: a prospective study. Am J Reprod Immunol. 2004; 52(4):276–83.

149. Wanachiwanawin D, Sutthent R, Chokephalbulkit K, Mahakittikun V, Ongrotchanakun J, Monkong N. *Toxoplasma gondii* antibodies in HIV and non-HIV infected Thai pregnant women. Asian Pac J Allergy Immunol. 2001; 19(4):291–3.

150. Tantivanich S, Amarapal P, Suphadtanaphongs W, Siripanth C, Sawatmongkonkun W. Prevalence of congenital cytomegalovirus and *Toxoplasma* antibodies in Thailand. [Southeast Asian J Trop Med Public Health.](https://www.ncbi.nlm.nih.gov/pubmed/11944699) 2001; 32(3):466–9.

151. Sukthana Y. Difference of *Toxoplasma gondii* antibodies between Thai and Austrian pregnant women. [Southeast Asian J Trop Med Public Health.](https://www.ncbi.nlm.nih.gov/pubmed/?term=Difference+of+Toxoplasma+gondii+antibodies+between+Thai+and+Austrian+pregnant+women.) 1999; 30(1):38–41.

152. Ashrafunnessa A, Khatun S, Islam MN, Huq T. Seroprevalence of *Toxoplasma* antibodies among the antenatal population in Bangladesh. J Obstetr Gynaecol Res. 1998; 24(2):115–9.

153. Taechowisan T, Sutthent R, Louisirirotchanakul S, Puthavathana P, Wasi C. Immune status in congenital infections by TORCH agents in pregnant Thais. Asian Pac J Allergy Immunol. 1997; 15(2):93–7.

154. Tanrıverdi Ç, Göktuğ BK, Alay H, Özkurt Z. Retrospective Evaluation of Anti-*Toxoplasma gondii* Antibody Among First Trimester Pregnant Women Admitted to Nenehatun Maternity Hospital between 2013-2017 in Erzurum. [Turkiye Parazitol Derg.](https://www.ncbi.nlm.nih.gov/pubmed/?term=Retrospective+Evaluation+of+Anti-Toxoplasma+gondii+Antibody+Among+First+Trimester+Pregnant+Women+Admitted+to+Nenehatun+Maternity+Hospital+between+2013-2017+in+Erzurum.) 2018; 42(2):101–105.

155. Sirin MC, Agus N, Yilmaz N, Bayram A, Derici YK, Samlioglu P, et al. Seroprevalence of *Toxoplasma gondii*, Rubella virus and Cytomegalovirus among pregnant women and the importance of avidity assays. Saudi Med J. 2017; 38(7):727–32.

156. Çetin M, Çetin Ş. Age-related prevalence of toxoplasmosis among pregnant women in Hatay: Estimation depending on model. Mikrobiyol Bull. 2017; 51(4):361–9.

157. Liassides M, Christodoulou V, Moschandreas J, Karagiannis C, Mitis G, Koliou M, et al. Toxoplasmosis in female high school students, pregnant women and ruminants in Cyprus. Trans R Soc Trop Med Hyg. 2016; 110(6):359–66.

158. Berghold C, Herzog SA, Jakse H, Berghold A. Prevalence and incidence of toxoplasmosis: a retrospective analysis of mother-child examinations, Styria, Austria, 1995 to 2012. Euro Surveill. 2016; 21(33).

159. Lange A, Thyrian J, Wetzka S, Flessa S, Hoffmann W, Zygmunt M, et al. The impact of socioeconomic factors on the efficiency of voluntary toxoplasmosis screening during pregnancy: a population-based study. BMC Pregnancy Childbirth. 2016; 16(1):197.

160. Billi P, Della Strada M, Pierro A, Semprini S, Tommasini N, Sambri V. Three-year retrospective analysis of the incidence of *Toxoplasma gondii* infection in pregnant women living in the Greater Romagna Area (northeastern Italy). Clin Microbiol Infect. 2016; 22(6):572. e1–e3.

161. Selek MB, Bektöre B, Baylan O, Özyurt M. Serological Investigation of *Toxoplasma gondii* on Pregnant Women and Toxoplasmosis Suspected Patients Between 2012-2014 Years on a Tertiary Training Hospital. [Turkiye Parazitol Derg.](https://www.ncbi.nlm.nih.gov/pubmed/?term=Retrospective+Evaluation+of+Anti-Toxoplasma+gondii+Antibody+Among+First+Trimester+Pregnant+Women+Admitted+to+Nenehatun+Maternity+Hospital+between+2013-2017+in+Erzurum.) 2015; 39(3):200–4.

162. Parlak M, Çim N, Erdin BN, Güven A, Bayram Y, Yıldızhan R. Seroprevalence of *Toxoplasma*, Rubella, and Cytomegalovirus among pregnant women in Van. [Turk J Obstet Gynecol.](https://www.ncbi.nlm.nih.gov/pubmed/?term=Seroprevalence+of+Toxoplasma%2C+Rubella%2C+and+Cytomegalovirus+among+pregnant+women+in+Van) 2015; 12(2):79–82.

163. Aynioglu A, Aynioglu O, Altunok ES. Seroprevalence of *Toxoplasma gondii*, rubella and Cytomegalovirus among pregnant females in north-western Turkey. Acta Clin Belg. 2015; 70(5):321–4.

164. Gundem NS, Agir MÇ. Investigation of seroprevalences of rubella and *Toxoplasma gondii* among pregnant women. Anatolian J Clin Invest. 2014; 8(4):152–157.

165. Karacan M, Batukan M, Çebi Z, Berberoglugil M, Levent S, Kır M, et al. Screening cytomegalovirus, rubella and *Toxoplasma* infections in pregnant women with unknown pre-pregnancy serological status. Arch Gynecol Obstetr. 2014; 290(6):1115–20.

166. Gencer M, Cevizci S, Saçar S, Vural A, Güngör ANÇ, Uysal A, et al. Evaluation of anti-*Toxoplasma gondii* antibody distribution and risk factors among pregnant women admitted to obstetrics polyclinic of Canakkale Onsekiz Mart University Hospital. Turkiye Parazitol Derg. 2014; 38(2):76–80.

167. Mumcuoglu I, Toyran A, Cetin F, Coskun FA, Baran I, Aksu N, et al. Evaluation of the toxoplasmosis seroprevalence in pregnant women and creating a diagnostic algorithm. Mikrobiyol Bul. 2014; 48(2):283–91.

168. Nowakowska D, Wujcicka W, Sobala W, Śpiewak E, Gaj Z, Wilczyński J. Age-associated prevalence of *Toxoplasma gondii* in 8281 pregnant women in Poland between 2004 and 2012. Epidemiol Infect. 2014; 142(3):656–61.

169. Uysal A, Cüce M, Tañer CE, Uysal F, Atalay S, Göl B, et al. Prevalence of congenital toxoplasmosis among a series of Turkish women. Rev Med Chile. 2013; 141(4):471–6.

170. Jerant-Patić V, Milošević V, Hrnjaković-Cvjetković I, Patić A, Stefan-Mikić S, Ristić M. *Toxoplasma gondii* infection in pregnant women. Med Pregl. 2013; 66(11-12):459–63.

171. Doğan K, Kafkaslı A, Karaman U, Atambay M, Karaoğlu L, Colak C. The rates of seropositivity and seroconversion of *Toxoplasma* infection in pregnant women. Mikrobiyol Bul. 2012; 46(2):290–4.

172. Dentico P, Volpe A, Putoto G, Ramadani N, Bertinato L, Berisha M, et al. Toxoplasmosis in Kosovo pregnant women. New Microbiol. 2011; 34(2):203–7.

173. Karabulut A, Polat Y, Türk M, Balci YI. Evaluation of rubella, *Toxoplasma gondii*, and cytomegalovirus seroprevalences among pregnant women in Denizli province. Turk J Med Sci. 2011; 41(1):159–64.

174. Sagel U, Krämer A, Mikolajczyk RT. Incidence of maternal *Toxoplasma* infections in pregnancy in Upper Austria, 2000-2007. BMC Infect Dis. 2011; 11(1):348.

175. De Paschale M, Agrappi C, Manco MT, Cerulli T, Clerici P. Implementation of screening for *Toxoplasma gondii* infection in pregnancy. J Clin Med Res. 2010; 2(3):112.

176. Maggi P, Volpe A, Carito V, Schinaia N, Bind S, Basho M, et al. Surveillance of toxoplasmosis in pregnant women in Albania. New Microbiol. 2009; 32(1):89–92.

177. Tamer GS, Dundar D, Caliskan E. Seroprevalence of *Toxoplasma gondii*, rubella and cytomegalovirus among pregnant women in western region of Turkey. Clin Invest Med. 2009; 32(1):E43.

178. Dolgikh T, Zapariĭ N, Kadtsyna T, Kalitin A. Epidemiological and clinicoimmunological monitoring of toxoplasmosis in the Omsk region. [Med Parazitol (Mosk).](https://www.ncbi.nlm.nih.gov/pubmed/?term=Epidemiological+and+clinicoimmunological+monitoring+of+toxoplasmosis+in+the+Omsk+region.) 2008;1:19–22.

179. Bartolomé Alvarez J, Martínez MS, Moreno LP, Lorente SO, Crespo MS. Prevalence and incidence in Albacete, Spain, of *Toxoplasma gondii* infection in women of childbearing age: Differences between immigrant and non-immigrant (2001-2007). [Rev Esp Salud Publica.](https://www.ncbi.nlm.nih.gov/pubmed/?term=Prevalence+and+incidence+in+Albacete%2C+Spain%2C+of+Toxoplasma+gondii+infection+in+women+of+childbearing+age%3A+Differences+between+immigrant+and+non-immigrant+(2001-2007).) 2008; 82(3):333–42.

180. De Paschale M, Agrappi C, Clerici P, Mirri P, Manco M, Cavallari S, et al. Seroprevalence and incidence of *Toxoplasma gondii* infection in the Legnano area of Italy. Clin Microbiol Infect. 2008; 14(2):186–9.

181. Ocak S, Zeteroglu S, Ozer C, Dolapcioglu K, Gungoren A. Seroprevalence of *Toxoplasma gondii*, rubella and cytomegalovirus among pregnant women in southern Turkey. Scand J Infect Dis. 2007; 39(3):231–4.

182. Nowakowska D, Stray‐Pedersen B, Śpiewak E, Sobala W, Małafiej E, Wilczyński J. Prevalence and estimated incidence of *Toxoplasma* infection among pregnant women in Poland: a decreasing trend in the younger population. Clin Microbiol Infect. 2006; 12(9):913–7.

183. Nash J, Chissel S, Jones J, Warburton F, Verlander N. Risk factors for toxoplasmosis in pregnant women in Kent, United Kingdom. Epidemiol Infect. 2005; 133(3):475–83.

184. Ertug S, Okyay P, Turkmen M, Yuksel H. Seroprevalence and risk factors for *Toxoplasma* infection among pregnant women in Aydin province, Turkey. BMC Public Health. 2005; 5(1):66.

185. Logar J, Šoba B, Premru‐Sršen T, Novak‐Antolic̆ Ž. Seasonal variations in acute toxoplasmosis in pregnant women in Slovenia. Clin Microbiol Infect. 2005; 11(10):852–5.

186. Antoniou M, Tzouvali H, Sifakis S, Galanakis E, Georgopoulou E, Liakou V, et al. Incidence of toxoplasmosis in 5532 pregnant women in Crete, Greece: management of 185 cases at risk. Eur J Obstetr Gynecol Reprod Biol. 2004; 117(2):138–43.

187. Harma M, Gungen N, Demir N. Toxoplasmosis in pregnant women in Sanliurfa, Southeastern Anatolia City, Turkey. J Egypt Soc Parasitol. 2004; 34(2):519–25.

188. Muñoz Batet C, Guardià CL, Juncosa TM, Viñas LD, Sierra MS, Sanfeliu IS, et al. Toxoplasmosis and pregnancy. Multicenter study of 16,362 pregnant women in Barcelona. Med Clín. 2004; 123(1):12–6.

189. Gutierrez-Zufiaurre N, Sanchez-Hernandez J, Munoz S, Marin R, Delgado N, Saenz M, et al. Seroprevalence of antibodies against *Treponema pallidum*, *Toxoplasma gondii*, rubella virus, hepatitis B and C virus, and HIV in pregnant women. [Enferm Infecc Microbiol Clin.](https://www.ncbi.nlm.nih.gov/pubmed/?term=Seroprevalence+of+antibodies+against+Treponema+pallidum%2C+Toxoplasma+gondii%2C+rubella+virus%2C+hepatitis+B+and+C+virus%2C+and+HIV+in+pregnant+women.) 2004; 22(9):512–6.

190. Ricci M, Pentimalli H, Thaller R, Rava L, Di Ciommo V. Screening and prevention of congenital toxoplasmosis: an effectiveness study in a population with a high infection rate. J Maternal-Fetal Neonatal Med. 2003; 14(6):398–403.

191. Logar J, Petrovec M, Novak-Antolic Z, Premru-Srsen T, Cizman M, Arnez M, et al. Prevention of congenital toxoplasmosis in Slovenia by serological screening of pregnant women. Scand J Infect Dis. 2002; 34(3):201–4.

192. Niemiec K, Raczyński P, Markiewicz K, Leibschang J, Ceran A. The prevalence of *Toxoplasma gondii* infection among 2016 pregnant women and their children in the Institute of Mother and Child in Warsaw. Wiad Parazytol. 2002; 48(3):293–9.

193. Vlaspolder F, Singer P, Smit A, Diepersloot R. Comparison of immulite with vidas for detection of infection in a low-prevalence population of pregnant women in The Netherlands. Clin Diag Lab Immunol. 2001; 8(3):552–5.

194. Evengård B, Petersson K, Engman M, Wiklund S, Ivarsson S, Teär-Fahnehjelm K, et al. Low incidence of *Toxoplasma* infection during pregnancy and in newborns in Sweden. Epidemiol Infect. 2001; 127(1):121–7.

195. Muñoz C, Izquierdo C, Parra J, Ginovart G, Margall N. Recommendation for prenatal screening for congenital toxoplasmosis. Eur J Clin Microbiol Infect Dis. 2000; 19(4):324–5.

196. Hejlicek K, Literak I, Vostalova E, Kresnicka J. *Toxoplasma gondii* antibodies in pregnant women in the Ceské Budĕjovice District. [Epidemiol Mikrobiol Imunol.](https://www.ncbi.nlm.nih.gov/pubmed/?term=Toxoplasma+gondii+antibodies+in+pregnant+women+in+the+Cesk%C3%A9+Bud%C4%95jovice+District) 1999; 48(3):102–5.

197. EvengÅrd B, Lilja G, Capraru T, Malm G, Kussofsky E, Öman H, et al. A retrospective study of seroconversion against *Toxoplasma gondii* during 3,000 pregnancies in Stockholm. Scand J Infect Dis.1999; 31(2):127–9.

198. Allain J-P, Palmer C, Pearson G. Epidemiological study of latent and recent infection by *Toxoplasma gondii* in pregnant women from a regional population in the UK. J Infect. 1998; 36(2):189–96.

199. Stojanović D. The effect of toxoplasmosis on occurrence of spontaneous abortions and anomalies in neonates in the Timok region. [Vojnosanit Pregl.](https://www.ncbi.nlm.nih.gov/pubmed/?term=The+effect+of+toxoplasmosis+on+occurrence+of+spontaneous+abortions+and+anomalies+in+neonates+in+the+Timok+region.) 1998; 55(2):151–9.

200. Jenum PA, Stray-Pedersen B, Melby KK, Kapperud G, Whitelaw A, Eskild A, et al. Incidence of *Toxoplasma gondii* infection in 35,940 pregnant women in Norway and pregnancy outcome for infected women. J Clin Microbiol. 1998; 36(10):2900–6.

201. Szénási Z, Ozsvar Z, Nagy E, Jeszenszky M, Szabo J, Gellen J, et al. Prevention of congenital toxoplasmosis in Szeged, Hungary. Int J Epidemiol. 1997; 26(2):428–35.

202. Buffolano W, Gilbert R, Holland F, Fratta D, Palumbo F, Ades A. Risk factors for recent *Toxoplasma* infection in pregnant women in Naples. Epidemiol Infect. 1996; 116(3):347–51.

203. Leone F, Allori B, Antognoli A, Catania S, Cerri B, Cicalini S, et al. Toxoplasmosis in pregnancy: research on 2295 women in Rome and its province. [Riv Eur Sci Med Farmacol.](https://www.ncbi.nlm.nih.gov/pubmed/?term=Toxoplasmosis+in+pregnancy%3A+research+on+2295+women+in+Rome+and+its+province) 1996; 18(5-6):191–5.

204. Lappalainen M, Koskela P, Koskiniemi M, Ämmälä P, Hiilesmaa V, Teramo K, et al. Toxoplasmosis acquired during pregnancy: improved serodiagnosis based on avidity of IgG. J Infect Dis. 1993; 167(3):691–7.

205. Valcavi P, Natali A, Soliani L, Montali S, Dettori G, Cheezi C. Prevalence of anti-*Toxoplasma gondii* antibodies in the population of the area of Parma (Italy). Eur J Epidemiol. 1995; 11(3):333–7.

206. Lebech M, Larsen S, Petersen E. Occurrence of toxoplasmosis in pregnant women in Denmark. A study of 5.402 pregnant women. Ugeskr Laeger. 1995; 157(38):5242–5.

207. Zadik P, Kudesia G, Siddons A. Low incidence of primary infection with *Toxoplasma* among women in Sheffield: a seroconversion study. [Br J Obstet Gynaecol.](https://www.ncbi.nlm.nih.gov/pubmed/?term=Low+incidence+of+primary+infection+with+Toxoplasma+among+women+in+Sheffield%3A+a+seroconversion+study) 1995; 102(8):608–10.

208. Zuber PL, Jacquier P, Hohlfeld P, Walker AM. *Toxoplasma* infection among pregnant women in Switzerland: a crosssectional evaluation of regional and age-specific lifetime average annual incidence. Am J Epidemiol. 1995; 141(7):659–66.

209. Logar J, Novak-Antolič Ž, Zore A. Serological screening for toxoplasmosis in pregnancy in Slovenia. Scand J Infect Dis.1995; 27(2):163–4.

210. Krausse T, Straube W, Wiersbitzky S, Hitz V, Kewitsch A. Screening for toxoplasmosis in pregnancy--a pilot program in Northeast Germany. [Geburtshilfe Frauenheilkd.](https://www.ncbi.nlm.nih.gov/pubmed/?term=Screening+for+toxoplasmosis+in+pregnancy--a+pilot+program+in+Northeast+Germany.) 1993; 53(9):613–8.

211. Henri T, Jacques S, Rene L. Twenty-two years screening for toxoplasmosis in pregnancy: Liege-Belgium. Scand J Infect Dis. 1992; 84:84–5.

212. Jaqueti J, Hernandez-Garcia R, Nicolas D, Martinez-Hernandez D, Navarro-Gallar F, Garcia-Esteban R. Serology against *Toxoplasma gondii* in pregnant women. Development of prevalence rates in the course of 4 years. Rev Clin Esp. 1991; 188(6):278–80.

213. Ahlfors K, Börjeson M, Huldt G, Forsberg E. Incidence of toxoplasmosis in pregnant women in the city of Malmö, Sweden. Scand J Infect Dis.1989; 21(3):315–21.

214. Conyn-van Spaendonck MAE. Prevention of congenital toxoplasmosis; experience in the Netherlands. Int Ophthalmol. 1989; 13(6):403–6.

215. Foulon W, Naessens A, Lauwers S, De FM, Amy J-J. Impact of primary prevention on the incidence of toxoplasmosis during pregnancy. Obstetr Gynecol. 1988; 72(3 Pt 1):363–6.

216. Jeannel D, Niel G, Costagliola D, Danis M, Traore BM, Gentilini M. Epidemiology of toxoplasmosis among pregnant women in the Paris area. Int J Epidemiol. 1988; 17(3):595–602.

217. Joss AW, Skinner LJ, Chatterton JM, Chisholm SM, Williams HD, Ho-Yen DO. Simultaneous serological screening for congenital cytomegalovirus and *Toxoplasma* infection. Public Health. 1988; 102(5):409–17.
