# Supplementary material for: Acute Toxoplasma infection in pregnant women worldwide: A systematic review and meta-analysis
Source: PLoS Negl Trop Dis. 2019 Oct 14;13(10):e0007807. doi: 10.1371/journal.pntd.0007807 (PMC6822777; doi:10.1371/journal.pntd.0007807)
Supplement: S2 Table — (DOCX) [file pntd.0007807.s003.docx]

**S2 Table.** Global, regional and national pooled prevalence of acute *Toxoplasma* infections (ATI) in pregnant women based on strict criteria^⁎^ (results from 82 datasets performed in 47 countries).

| WHO regions/ country | Number of datasets | Number of pregnant women with ATI/ number of pregnant women screened | Prevalence of ATI by simple pooling  % (95% CI) | Prevalence of ATI by random effect model meta-analysis  % (95% CI) | Heterogeneity *I*^2^ (%) |
| --- | --- | --- | --- | --- | --- |
| Global | **82** | **4,035/733,809** | **0.5 (0.49-0.50)** | **0.6 (0.4-0.7)** | **98.1** |
| Middle East and north Africa | **4** | 109/5073 | 2.1 (2.04-2.15) | **1.9 (1.0-3.0)** | **78.5** |
| Egypt | 1 | 1/323 | 0.3 (0.0-1.7) | 0.3 (0.0-1.7) | NA |
| Lebanon | 1 | 46/2456 | 1.9 (1.4-2.5) | 1.9 (1.4-2.5) | NA |
| Kuwaiti | 1 | 9/224 | 4.0 (1.9-7.5) | 4.0 (1.9-7.5) | NA |
| Tunisia | 1 | 53/2070 | 2.6 (1.9-3.3) | 2.6 (1.9-3.3) | NA |
| African region | **9** | **145/2998** | **4.8 (4.6-4.9)** | **2.2 (0.2-6.1)** | **96.3** |
| Senegal | 2 | 108/1050 | 10.2 (8.4-12.1) | 10.2 (8.4-12.1) | 0.0 |
| Burkina Faso | 1 | 0/316 | 0.0 (0.00-1.2) | 0.0 (0.00-1.2) | NA |
| Nigeria | 1 | 26/360 | 7.2 (4.8-10.4) | 7.2 (4.8-10.4) | NA |
| Algeria | 1 | 9/143 | 6.3 (2.9-11.6) | 6.3 (2.9-11.6) | NA |
| Benin | 1 | 0/283 | 0.0 (0.0-1.3) | 0.0 (0.0-1.3) | NA |
| Ghana | 1 | 0/168 | 0.1 (0.0-2.2) | 0.1 (0.0-2.2) | NA |
| Angola | 1 | 0/300 | 0.0 (0.0-1.2) | 0.0 (0.0-1.2) | NA |
| Congo | 1 | 2/378 | 0.5 (0.1-1.9) | 0.5 (0.1-1.9) | NA |
| Western Pacific Region | **4** | **143/7595** | **1.8 (1.76-1.83)** | **0.6 (0.1-3.2)** | **98.1** |
| Malaysia | 2 | 1/500 | 0.2 (0.18-0.21) | 0.1 (0.0-0.8) | 0.0 |
| China | 1 | 139/4126 | 3.4 (2.8-4.0) | 3.4 (2.8-4.0) | NA |
| Japan | 1 | 3/2969 | 0.1 (0.0-0.3) | 0.1 (0.0-0.3) | NA |
| Latin America & Caribbean region | **10** | **420/83,440** | **0.5 (0.49-0.5)** | **0.5 (0.2-0.9)** | **95.6** |
| Brazil | 7 | 273/67524 | 0.4 (0.39-0.4) | 0.3 (0.1-0.7) | 95.5 |
| Venezuela | 1 | 10/678 | 1.5 (0.7-2.7) | 1.5 (0.7-2.7) | NA |
| Argentina | 1 | 121/13632 | 0.9 (0.7-1.1) | 0.9 (0.7-1.1) | NA |
| Cuba | 1 | 16/1606 | 1.0 (0.6-1.6) | 1.0 (0.6-1.6) | NA |
| South-East Asian Region | **4** | **8/1358** | 0.58 (0.55-0.6) | **0.5 (0.1-1.3)** | **43.1** |
| India | 2 | 5/944 | 0.4 (0.1-1.0) | 0.4 (0.1-1.0) | 0.0 |
| Thailand | 1 | 3/199 | 1.5 (0.3-4.3) | 1.5 (0.3-4.3) | NA |
| Myanmar | 1 | 0/215 | 0.0 (0.0-1.7) | 0.0 (0.0-1.7) | NA |
| Europe region | **51** | **3210/633,345** | **0.5 (0.49-0.5)** | **0.4 (0.3-0.6)** | **98.5** |
| Turkey | 10 | 144/68,306 | 0.2 (0.19-0.2) | 0.1 (0.0-0.3) | 95.1 |
| Italy | 5 | 288/56,660 | 0.5 (0.49-0.5) | 0.5 (0.1-1.3) | 98.7 |
| Spain | 4 | 47/25,254 | 0.18 (0.17-0.18) | 0.2 (0.0-0.6) | 93.7 |
| Poland | 3 | 79/15,213 | 0.5 (0.3-0.7) | 0.5 (0.3-0.7) | 0.0 |
| Sweden | 3 | 28/48,423 | 0.05 (0.49-0.5) | 0.1 (0.0-0.3) | 0.0 |
| Slovenia | 3 | 354/82,304 | 0.4 (0.3-0.6) | 0.4 (0.3-0.6) | 0.0 |
| United Kingdom | 2 | 3/3518 | 0.1 (0.0-0.2) | 0.1 (0.0-0.2) | 0.0 |
| Austria | 2 | 946/166,732 | 0.5 (0.5-0.5) | 0.5 (0.5-0.5) | 0.0 |
| Germany | 2 | 28/9757 | 0.2 (0.1-0.4) | 0.3 (0.2-0.4) | 0.0 |
| Netherland | 2 | 58/28,549 | 0.2 (0.1-0.3) | 0.2 (0.1-0.2) | 0.0 |
| Belgium | 2 | 286/27,450 | 1.0 (0.9-1.1) | 1.0 (0.9-1.1) | 0.0 |
| Portugal | 1 | 0/155 | 0.0 (0.0-2.0) | 0.0 (0.0-2.4) | NA |
| Kosovo | 1 | 4/334 | 1.1 (0.3-3.0) | 1.2 (0.3-3.0) | NA |
| Albania | 1 | 2/496 | 0.4 (0.4-1.4) | 0.4 (0.0-1.4) | NA |
| Greece | 1 | 185/5,532 | 3.3 (2.8-3.8) | 3.3 (2.9-3.9) | NA |
| Denmark | 1 | 35/5,402 | 0.6 (0.4-0.9) | 0.6 (0.5-0.9) | NA |
| Hungary | 1 | 78/17,735 | 0.4 (0.3-0.6) | 0.4 (0.3-0.5) | NA |
| Russia | 1 | 393/9,365 | 4.1 (3.7-4.6) | 4.2 (3.8-4.6) | NA |
| Czech Republic | 1 | 20/1409 | 1.4 (0.8-2.2) | 1.4 (0.9-2.2) | NA |
| France | 1 | 35/2,216 | 1.6 (1.1-2.2) | 1.6 (1.1-2.2) | NA |
| Norway | 1 | 47/32,033 | 0.1 (0.1-0.2) | 0.1 (0.1-0.2) | NA |
| Finland | 1 | 25/16,733 | 0.1 (0.0-0.2) | 0.1 (0.1-0.2) | NA |
| Scotland | 1 | 10/4,548 | 0.2 (0.1-0.4) | 0.2 (0.1-0.4) | NA |
| Switzerland | 1 | 115/5,221 | 2.2 (1.8-2.6) | 2.2 (1.8-2.6) | NA |

**Abbreviations:** NA: not applicable

WHO regions are sorted according to prevalence rates

Countries are sorted according to number of studies included

⁎ In this analysis, we excluded studies that had just IgG positive and IgM positive, and only studies that had seroconversion OR IgG positive/IgM positive and low IgG avidity results were included to estimate the prevalence of ATI in pregnant women.
