# Supplementary figures and images for: Acute Toxoplasma infection in pregnant women worldwide: A systematic review and meta-analysis
Source: PLoS Negl Trop Dis. 2019 Oct 14;13(10):e0007807. doi: 10.1371/journal.pntd.0007807 (PMC6822777; doi:10.1371/journal.pntd.0007807)

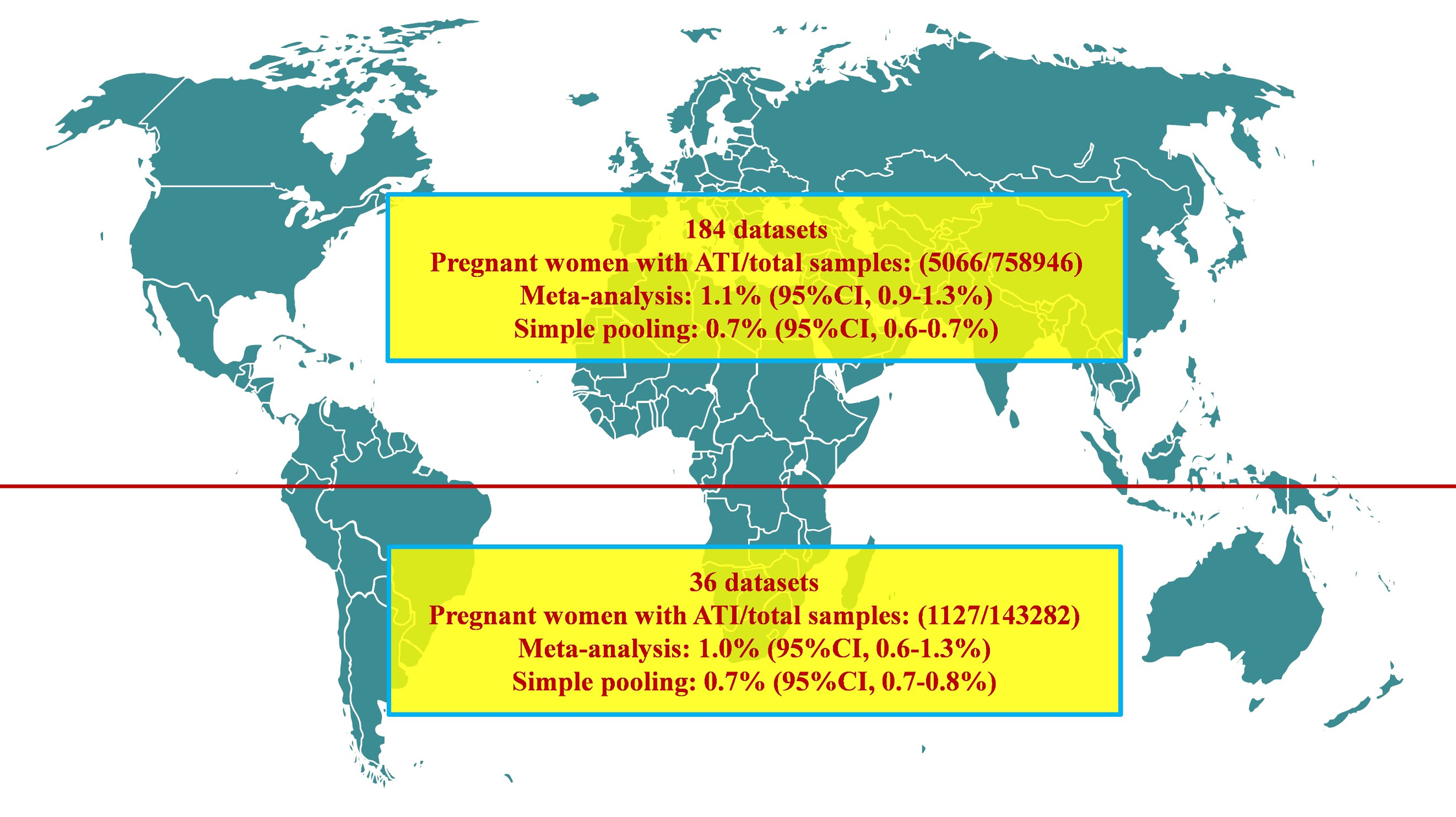

Supplement: S1 Fig — Abbreviations: CI, confidence interval; ATI, acute Toxoplasma Infections. (TIF) [file pntd.0007807.s004.tif]

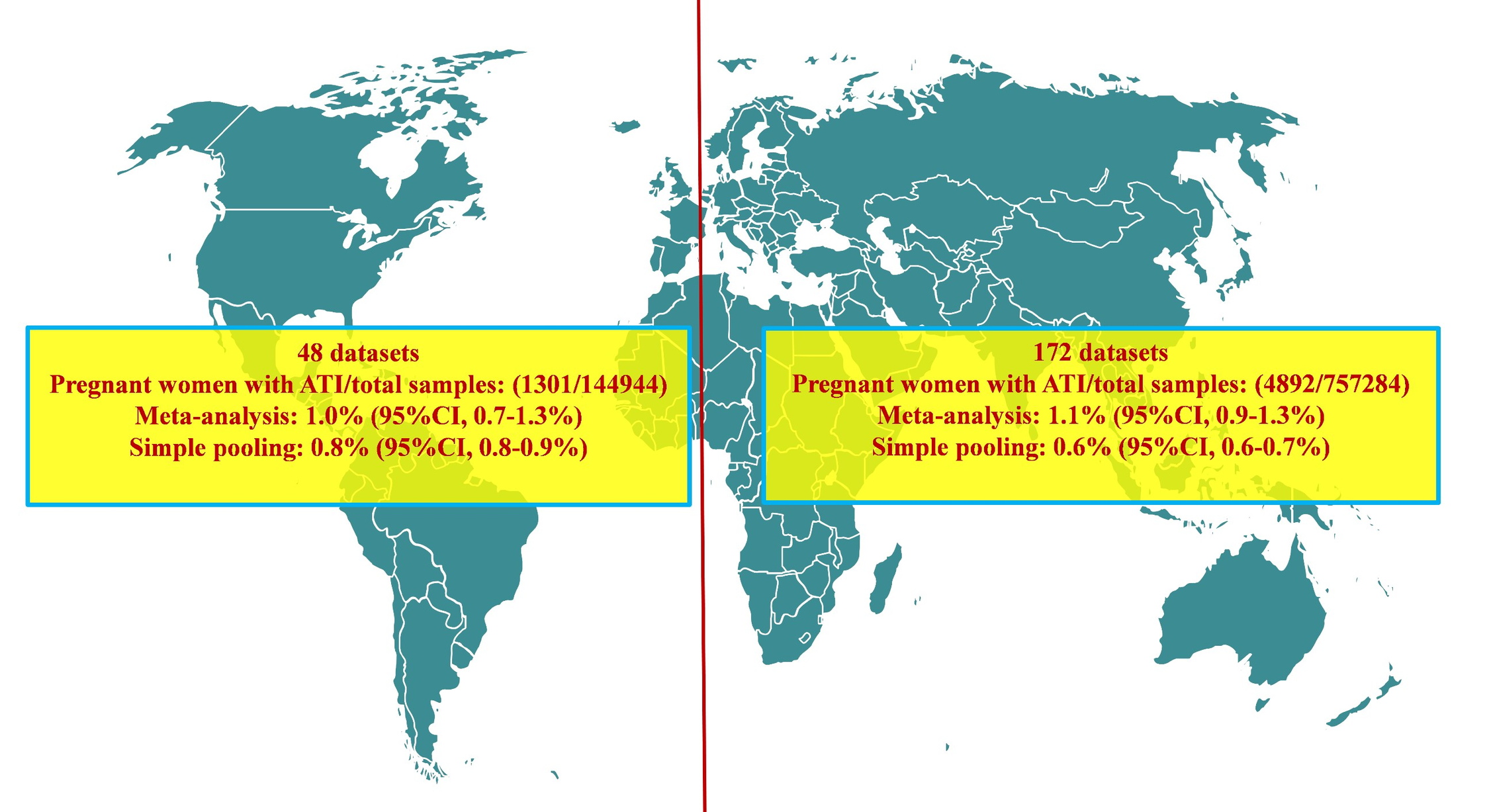

Supplement: S2 Fig — Abbreviations: CI, confidence interval; ATI, acute Toxoplasma Infections. (TIF) [file pntd.0007807.s005.tif]

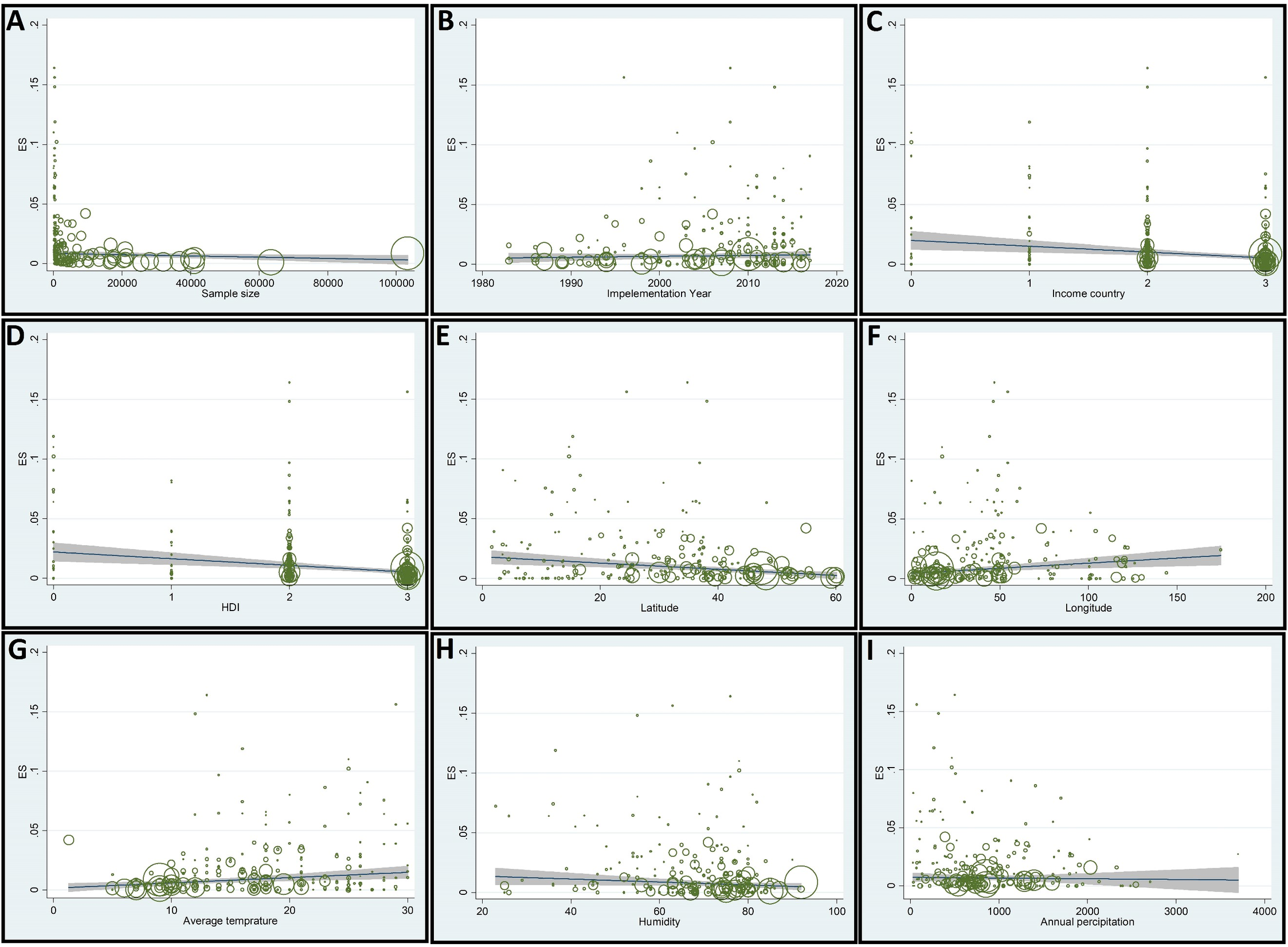

Supplement: S3 Fig — Ecological linear meta-regression analyses of the prevalence of acute Toxoplasma infections (ATI) in pregnant women according to: (panel A) sample size showing a non-statistically significant downward trend in prevalence with increasing sample size (C = -5. 79e-08; P-value = 0.1); (panel B) implementation years of screening showing a non-statistically significant upward trend in prevalence in more recent years (C = 0.0007; P-value = 0.57); (panel C) country’s income level showing a showing a statistically significant downward trend in prevalence in countries with higher level of income (C = -0.005; P-value = 0.03); (panel D) human development index (HDI) showing a statistically significant downward trend in prevalence in countries with higher levels of HDI (C = -0.005; P-value = 0.006); (panel E) geographical latitude a showing a statistically significant downward trend in prevalence with increasing geographical latitude (C = -0.00026, P-value = 0.005); (panel F) geographical longitude a showing a statistically significant upward trend in prevalence with increasing geographical longitude (C = 0.0008, P-value = 0.02); (panel G) the mean temperature showing a statistically significant upward trend in prevalence with increasing mean temperature (C = 0.0004; P-value = 0.02); (panel H) the relative humidity showing a non-statistically significant downward trend in prevalence in areas with higher relative humidity (C = -0.0001, P-value = 0.18); (panel I) the annual precipitation showing a non-statistically significant downward trend in prevalence with increasing rate of precipitation (C = -6.41, P-value = 0.81). Abbreviations: C, coefficient; ES, effect size (Prevalence of ATI). (TIF) [file pntd.0007807.s006.tif]
