# Supplementary material for: Acute Toxoplasma infection in pregnant women worldwide: A systematic review and meta-analysis
Source: PLoS Negl Trop Dis. 2019 Oct 14;13(10):e0007807. doi: 10.1371/journal.pntd.0007807 (PMC6822777; doi:10.1371/journal.pntd.0007807)
Supplement: S1 Text — (DOCX) [file pntd.0007807.s007.docx]

**S1 Text.** Details of the databases searches

**Scopus=2204**

## 1. TITLE-ABS-KEY (("toxoplasma gondii" OR "toxoplasma infection" OR "toxoplasmosis" )  AND  ("acute infection" OR "primary infection"  OR  "seroconversion" )  AND  ("seroprevalence" OR  "prevalence"  OR  "incidence" )  AND( pregnant  AND women))=171

## 2. TITLE-ABS-KEY (("toxoplasma gondii") AND (prevalence) AND ("pregnant women"))=347

## 3. TITLE-ABS-KEY (("toxoplasma gondii" OR "toxoplasmosis" OR "toxoplasma infection") AND ("pregnant women"))=1686

**PubMed searching results=2413**

## 1. ("toxoplasmosis"[MeSH Terms] OR "toxoplasmosis"[All Fields]) AND ("epidemiology"[Subheading] OR "epidemiology"[All Fields] OR "prevalence"[All Fields] OR "prevalence"[MeSH Terms]) AND ("pregnant women"[MeSH Terms] OR ("pregnant"[All Fields] AND "women"[All Fields]) OR "pregnant women"[All Fields]) AND ("1988/01/01"[PDAT] : "2018/11/30"[PDAT])**= 568**

## 2. (("toxoplasma"[MeSH Terms] OR "toxoplasma"[All Fields]) AND ("infection"[MeSH Terms] OR "infection"[All Fields])) AND ("pregnant women"[MeSH Terms] OR ("pregnant"[All Fields] AND "women"[All Fields]) OR "pregnant women"[All Fields]) AND ("1988/01/01"[PDAT] : "2018/11/30"[PDAT])**= 755**

## 4. ("toxoplasma gondii"[All Fields] OR "toxoplasmosis"[All Fields]) AND ("acute infection"[All Fields] OR "primary infection"[All Fields] OR "seroconversion"[All Fields]) AND ("prevalence"[All Fields] OR "seroprevalence"[All Fields] OR "seroepidemiology"[All Fields]) AND ("pregnant women"[All Fields] OR "pregnancy"[All Fields]) AND ("1988/01/01"[PDAT] : "2018/11/30"[PDAT])=**128**

## 4. (("toxoplasma"[MeSH Terms] OR "toxoplasma"[All Fields]) AND ("infection"[MeSH Terms] OR "infection"[All Fields])) AND ("epidemiology"[Subheading] OR "epidemiology"[All Fields] OR "epidemiology"[MeSH Terms]) AND ("pregnancy"[MeSH Terms] OR "pregnancy"[All Fields]) AND ("pregnant women"[MeSH Terms] OR ("pregnant"[All Fields] AND "women"[All Fields]) OR "pregnant women"[All Fields]) AND ("1988/01/01"[PDAT] : "2018/11/30"[PDAT])= **344**

## 5. (("toxoplasmosis"[MeSH Terms] OR "toxoplasmosis"[All Fields]) OR "Toxoplasma gondii"[All Fields]) AND (("epidemiology"[Subheading] OR "epidemiology"[All Fields] OR "prevalence"[All Fields] OR "prevalence"[MeSH Terms]) OR ("epidemiology"[Subheading] OR "epidemiology"[All Fields] OR "incidence"[All Fields] OR "incidence"[MeSH Terms])) AND ("pregnant women"[MeSH Terms] OR ("pregnant"[All Fields] AND "women"[All Fields]) OR "pregnant women"[All Fields]) AND ("1988/01/01"[PDAT] : "2018/11/30"[PDAT])=**618**

## 
